# Prolyl Carboxypeptidase Mediates the C-Terminal Cleavage of (Pyr)-Apelin-13 in Human Umbilical Vein and Aortic Endothelial Cells

**DOI:** 10.3390/ijms22136698

**Published:** 2021-06-22

**Authors:** Emilie De Hert, An Bracke, Isabel Pintelon, Eline Janssens, Anne-Marie Lambeir, Pieter Van Der Veken, Ingrid De Meester

**Affiliations:** 1 Laboratory of Medical Biochemistry, Faculty of Pharmaceutical, Biomedical and Veterinary Sciences, University of Antwerp, 2610 Wilrijk, Belgium; emilie.dehert@uantwerpen.be (E.D.H.); an.bracke@uantwerpen.be (A.B.); eline.janssens2@uantwerpen.be (E.J.); anne-marie.lambeir@uantwerpen.be (A.-M.L.); 2 Laboratory of Cell Biology and Histology, Faculty of Pharmaceutical, Biomedical and Veterinary Sciences; Faculty of Medicine and Health Sciences, University of Antwerp, 2610 Wilrijk, Belgium; isabel.pintelon@uantwerpen.be; 3 Laboratory of Experimental Medicine and Pediatrics, Faculty of Medicine and Health Sciences, University of Antwerp, 2610 Wilrijk, Belgium; 4 Laboratory of Medicinal Chemistry, Faculty of Pharmaceutical, Biomedical and Veterinary Sciences, University of Antwerp, 2610 Wilrijk, Belgium; pieter.vanderveken@uantwerpen.be

**Keywords:** angiotensin converting enzyme 2, human endothelial cells, prolyl carboxypeptidase, (pyr)-apelin-13

## Abstract

The aim of this study was to investigate the C-terminal cleavage of (pyr)-apelin-13 in human endothelial cells with respect to the role and subcellular location of prolyl carboxypeptidase (PRCP). Human umbilical vein and aortic endothelial cells, pre-treated with prolyl carboxypeptidase-inhibitor compound 8o and/or angiotensin converting enzyme 2 (ACE2)-inhibitor DX600, were incubated with (pyr)-apelin-13 for different time periods. Cleavage products of (pyr)-apelin-13 in the supernatant were identified by mass spectrometry. The subcellular location of PRCP was examined via immunocytochemistry. In addition, PRCP activity was measured in supernatants and cell lysates of LPS-, TNFα-, and IL-1β-stimulated cells. PRCP cleaved (pyr)-apelin-13 in human umbilical vein and aortic endothelial cells, while ACE2 only contributed to this cleavage in aortic endothelial cells. PRCP was found in endothelial cell lysosomes. Pro-inflammatory stimulation induced the secretion of PRCP in the extracellular environment of endothelial cells, while its intracellular level remained intact. In conclusion, PRCP, observed in endothelial lysosomes, is responsible for the C-terminal cleavage of (pyr)-apelin-13 in human umbilical vein endothelial cells, while in aortic endothelial cells ACE2 also contributes to this cleavage. These results pave the way to further elucidate the relevance of the C-terminal Phe of (pyr)-apelin-13.

## 1. Introduction

Prolyl carboxypeptidase (PRCP, angiotensinase C, EC 3.4.16.2) is a serine protease known for its role in the alternative renin-angiotensin system and plasma kallikrein-kinin system [1,2,3]. Soon after its discovery as des-Arg^9^-bradykinin-, angiotensin II-, and III-cleaving enzyme, it was clear that PRCP cleaves C-terminal amino acids from peptides with a general structure of R1-Pro-R2 [1,4]. PRCP has been implicated in the pathophysiology of hypertension and inflammation [5,6,7,8,9]. However, since Wallingford et al. discovered that PRCP is responsible for inactivating the anorexigenic peptide alpha-melanocyte stimulating hormone 1–13 (α-MSH 1–13), PRCP mainly received interest for its role in metabolic disorders [10,11,12,13,14]. Both non-brain-penetrating and centrally active PRCP-inhibitors showed body weight reduction in diet-induced obesity in mice [15,16]. These findings suggested that besides the centrally active α-MSH 1–13, PRCP can also truncate peripherally acting peptides involved in body weight regulation. The search for other candidate substrates led to the identification of pyroglutamated apelin-13 ((pyr)-apelin-13) as a novel in vitro substrate for PRCP [17].

(Pyr)-apelin-13 and the other apelin isoforms, including apelin-36, apelin-17, and apelin-13, originating from the same precursor preproapelin, are the endogenous ligands of the G-protein-coupled receptor APJ [18]. (Pyr)-apelin-13 is the most abundant isoform in human plasma and cardiac tissue and is therefore used in this study [19,20]. Apelin-APJ signaling is important for cardiovascular regulation [21,22,23,24,25]. In addition, apelin is an adipokine, a bioactive mediator secreted by adipocytes and therefore, apelin-mediated signaling pathways are promising therapeutic targets in different metabolic pathologies [23,24,26].

Although (pyr)-apelin-13 has increased stability and a longer plasma half-life in comparison with the non-pyroglutamated isoforms, it still has a short half-life of only minutes [20,27]. Angiotensin-converting enzyme 2 (ACE2, EC 3.4.17.23) was always considered to be the only enzyme responsible for the hydrolysis of the C-terminal Phe of (pyr)-apelin-13 [28,29,30], until we identified PRCP as an apelin-cleaving enzyme in 2016, during the search for new PRCP-substrates based on amino acid sequence [17]. Although many efforts were made to elucidate the importance of this C-terminal Phe, a lot of inconsistencies still exist on the exact role of this amino acid and the enzymes responsible for the C-terminal truncation [31,32,33,34,35,36,37,38,39]. Therefore, studying the C-terminal cleavage of (pyr)-apelin-13 still remains important.

As both PRCP and the APJ receptor are reported to be expressed on the plasma membrane of endothelial cells and intracellularly in vesicles and as apelin plays an important role in endothelial cell function regulation, these cells are well suited to further elucidate the role of PRCP in apelin cleavage [21,40,41,42]. Shariat-Madar et al. investigated the localization of PRCP in human umbilical vein endothelial cells (HUVEC) and reported PRCP to be present on the cell membrane and in lysosomes [40], but the data on PRCP’s subcellular location are scarce and uncertain. The fact that PRCP is found extracellularly as soluble protein in urine, plasma, and synovial fluid, and as part of the secretome of human endothelial cells [43,44], can indicate that PRCP is transported via the endosomal pathway and secreted in the extracellular environment. Therefore, a thorough understanding of PRCP’s location in and on endothelial cells will provide a better insight into PRCP’s role in the cleavage of circulating peptides.

As a previous study revealed that PRCP mRNA expression was upregulated in lipopolysaccharide (LPS)-activated endothelial cells [45], it is also of interest to further elucidate the influence of pro-inflammatory stimulation on PRCP’s activity and location in endothelial cells.

In the current study, we focus on the C-terminal cleavage of (pyr)-apelin-13 and the role of PRCP herein. To this end, we investigated the cleavage of (pyr)-apelin-13 in HUVEC and human aortic endothelial cells (HAoEC) and analyzed the contribution of PRCP and ACE2 by use of the selective inhibitors compound 8o and DX600 [46,47]. Furthermore, we investigated the subcellular location of PRCP by immunofluorescence and tested the influence of pro-inflammatory stimuli on PRCP’s location and secretion in endothelial cells.

Our results demonstrate that (pyr)-apelin-13 is cleaved at its C-terminus in HUVEC and HAoEC. The enzymes involved differ between the two cell types. In HUVEC, the cleavage is mediated only by PRCP, while in HAoEC, also ACE2 contributes to this cleavage. Moreover, we confirm that PRCP is found in endothelial lysosomes. Pro-inflammatory stimulation induces PRCP secretion, while its activity level inside the cells remains constant.

## 2. Results

### 2.1. Cleavage of (Pyr)-Apelin-13 to (Pyr)-Apelin-13_(1–12)_ Is PRCP-Dependent in HUVEC and PRCP- and ACE2-Dependent in HAoEC

**Figure 1:**
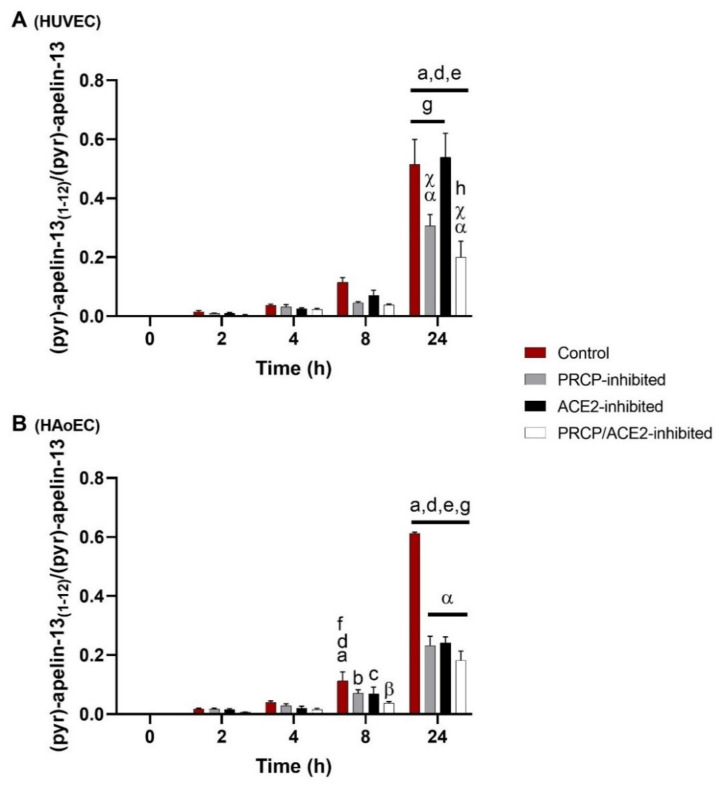
Cleavage of (pyr)-apelin-13 to (pyr)-apelin-13_(1–12)_ is PRCP-dependent in HUVEC and PRCP- and ACE2-dependent in HAoEC. C-terminal cleavage of (pyr)-apelin-13 to (pyr)-apelin-13_(1–12)_ in HUVEC (**A**) and HAoEC (**B**) (*n* = 4 per group), expressed as the ratio of the peak intensity of (pyr)-apelin-13_(1–12)_ to the peak intensity of (pyr)-apelin-13 measured in the supernatant. Two-way ANOVA analysis revealed a significant interaction between the effects of treatment duration and treatment option on the ratio of the peak intensities (*p* < 0.001) in both types of endothelial cells. Results are reported as mean ± SEM. Significant p-values are indicated on the graph (a: different from 0 h, *p* < 0.001; b: different from 0 h, *p* < 0.01; c: different from 0 h, *p* < 0.05; d: different from 2 h, *p* < 0.001; e: different from 4 h, *p* < 0.001; f: different from 4 h, *p* < 0.01; g: different from 8 h, *p* < 0.001; h: different from 8 h, *p* < 0.01; α: different from control, *p* < 0.001; β: different from control, *p* < 0.01; χ: different from ACE2-inhibited group, *p* < 0.001; within treatment group and time point group respectively).

C-terminal cleavage of (pyr)-apelin-13 to (pyr)-apelin-13_(1–12)_ in HUVEC and HAoEC (*n* = 4 per group) is shown in Figure 1 and is expressed as the ratio of the peak intensity of (pyr)-apelin-13_(1–12)_ to the peak intensity of (pyr)-apelin-13 measured in the supernatant. The cleavage is significant after 24 h within all treatment groups in both types of endothelial cells, but is already observable after 8 h in control, PRCP-inhibited, and ACE2-inhibited HAoEC. Contrary to the cell supernatants, none of the apelin forms could be detected in the cell lysate under our experimental conditions. 

Significantly less (pyr)-apelin-13_(1–12)_ was detected in PRCP-inhibited or PRCP/ACE2-inhibited HUVEC after 24 h compared with control or ACE2-inhibited HUVEC. In the supernatants of all inhibitor-treated HAoEC, significantly less (pyr)-apelin-13_(1–12)_ was observed after 24 h compared with the control group. 

The determination of additional cleavage sites revealed (pyr)-apelin-13_(1-6)_ (**m/z** 739.4) as a product of (pyr)-apelin-13 cleavage in HAoEC. In HUVEC, this product could not be detected. The cleavage is significant from 8 h onwards within all treatment groups, but none of the studied enzymes were involved (data not shown). Other fragments with *m/z* values corresponding to possible cleavage products of (pyr)-apelin-13 could be detected in the cellular environment of both types of endothelial cells, but in lower amounts, and their identity could not be confirmed by MS/MS analysis.

### 2.2. α-MSH 1–13 Is Cleaved at Its C-Terminus in Function of Time in HUVEC and HAoEC

**Figure 2:**
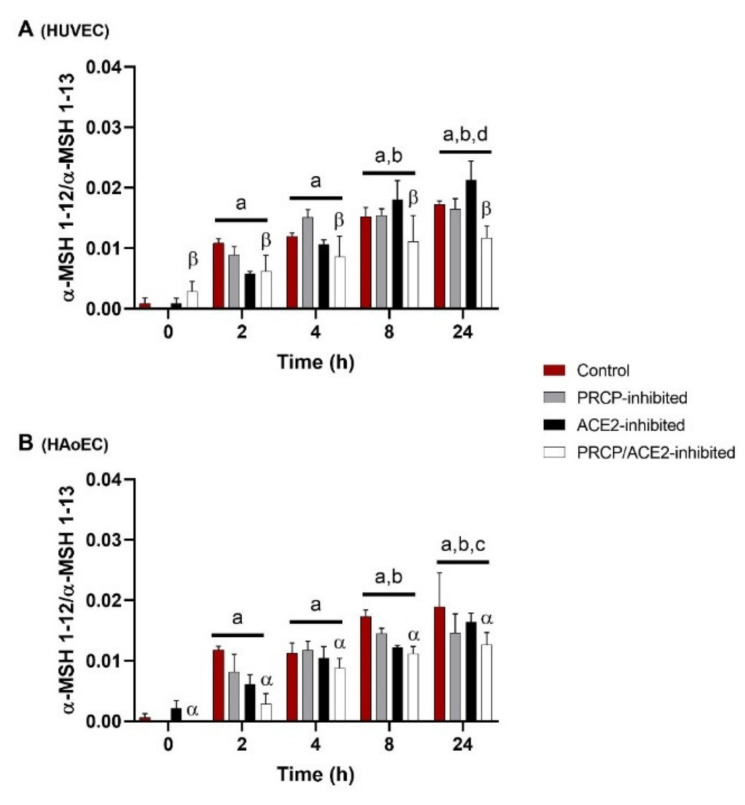
α-MSH 1–13 is cleaved at its C-terminus in function of time in HUVEC and HAoEC. C-terminal cleavage of α-MSH 1–13 to α-MSH 1–12 in HUVEC (**A**) and HAoEC (**B**) (*n* = 4 per group), expressed as the ratio of the peak intensity of α-MSH 1–12 to the peak intensity of α-MSH 1–13 measured in the supernatant. Two-way ANOVA analysis showed no significant interaction between the effects of treatment duration and treatment option on the ratio of the peak intensities for the C-terminal cleavage of α-MSH 1–13 (*p* = 0.159 for HUVEC and *p* = 0.596 for HAoEC) in both types of endothelial cells. For the treatment groups (*p* = 0.029 for HUVEC and *p* < 0.001 for HAoEC) and the time point groups (*p* < 0.001), significant main effects were reported. Results are reported as mean ± SEM. Significant *p*-values are indicated on the graph (a: different from 0 h, *p* < 0.001; b: different from 2 h, *p* < 0.001; c: different from 4 h, *p* < 0.01; d: different from 4 h, *p* < 0.05; α: different from control, *p* < 0.01; β: different from PRCP-inhibited group, *p* < 0.01; overall effect).

C-terminal cleavage of α-MSH 1–13 to α-MSH 1–12 in HUVEC and HAoEC (*n* = 4 per group) is shown in Figure 2 and is expressed as the ratio of the peak intensity of α-MSH 1–12 to the peak intensity of α-MSH 1–13 measured in the supernatant. α-MSH 1–13 is cleaved at its C-terminus in function of time. An overall significant difference was observed between PRCP-inhibited HUVEC and PRCP/ACE2-inhibited HUVEC and between control and PRCP/ACE2-inhibited HAoEC. α-MSH 1-10 (*m/z* 1340.5) was also detected in the extracellular environment of HUVEC and HAoEC. This cleavage also occurred in function of time, but no effect of the inhibitors was seen (data not shown). Other fragments with *m/z* values corresponding to possible cleavage products of α-MSH 1–13 could be detected in the cellular environment of both types of endothelial cells, but in lower amounts, and their identity could not be confirmed by MS/MS analysis.

### 2.3. PRCP Is Observed in Lysosomes of HUVEC and HAoEC

**Figure 3:**
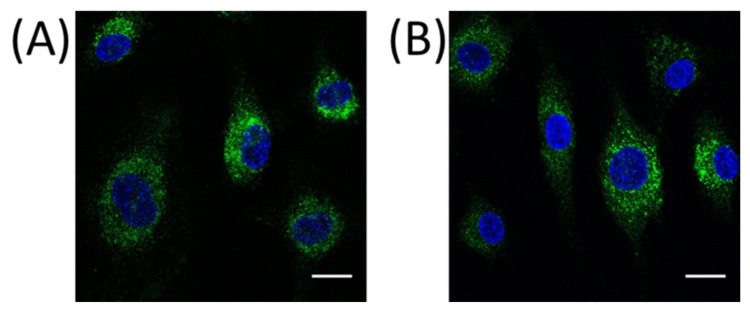
PRCP is observed in cytoplasmatic vesicular structures of HUVEC and HAoEC. Immunofluorescent staining of PRCP in HUVEC (**A**) and HAoEC (**B**). Cells were fixed with 4% paraformaldehyde (PFA), permeabilized with 0.1% Triton X-100 and stained for PRCP (green) and DAPI (nuclear marker, blue). (bar = 20 µm).

**Figure 4:**
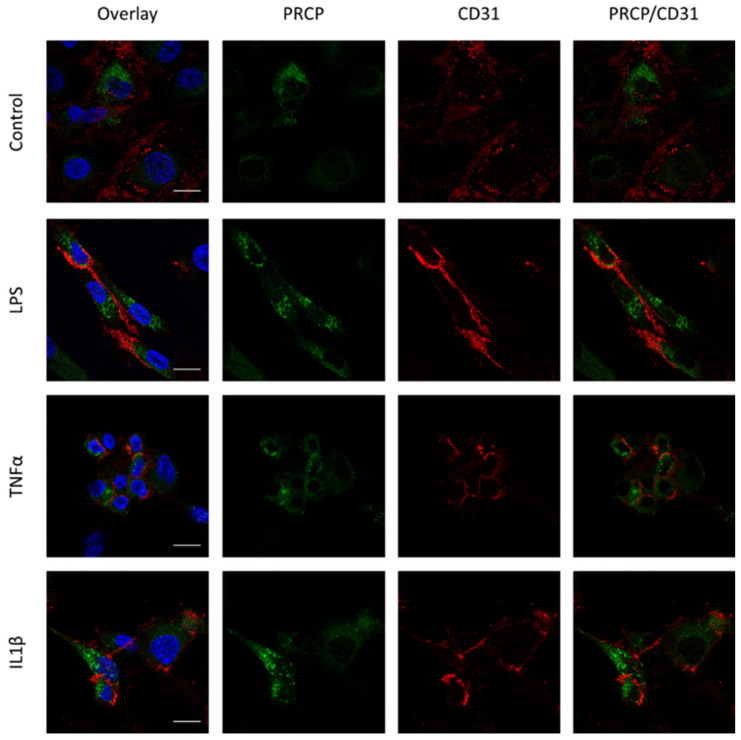
Overlap between PRCP and CD31 is not observed in HUVEC. Double staining of PRCP and CD31 in control, LPS-, TNFα- and IL-1β-stimulated permeabilized HUVEC. Cells were incubated for 16 h with the different stimuli, fixed with 4% PFA, permeabilized with 0.1% Triton X-100 and stained for PRCP (green), CD31 (red), and DAPI (nuclear marker, blue). Representative images of three independent experiments. Non-permeabilized cells showed comparable results. (bar = 20 µm).

**Figure 5:**
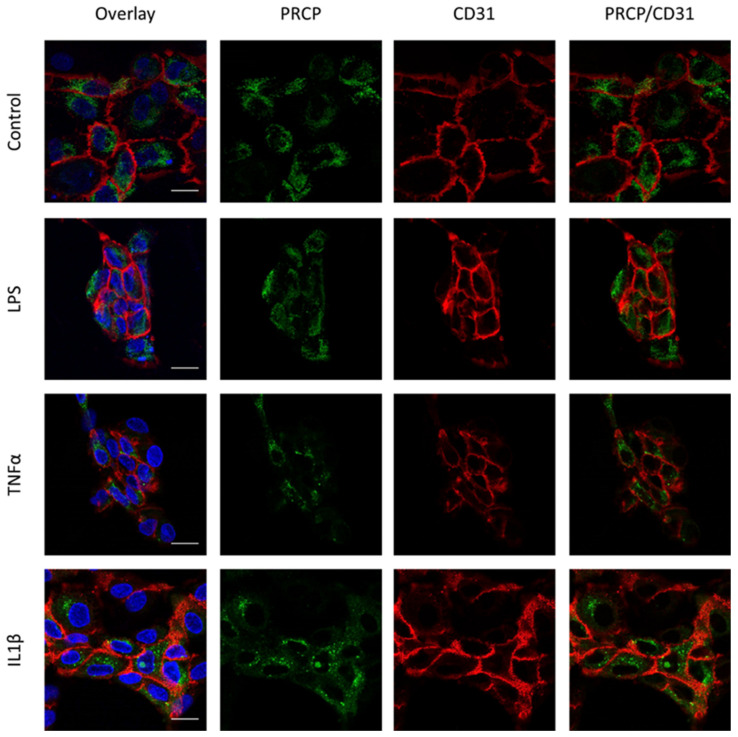
Overlap between PRCP and CD31 is not observed in HAoEC. Double staining of PRCP and CD31 in control, LPS-, TNFα-, and IL-1β-stimulated permeabilized HAoEC. Cells were incubated for 16 h with the different stimuli, fixed with 4% PFA, permeabilized with 0.1% Triton X-100 and stained for PRCP (green), CD31 (red), and DAPI (nuclear marker, blue). Representative images of three independent experiments. Non-permeabilized cells showed comparable results. (bar = 20 µm).

**Figure 6:**
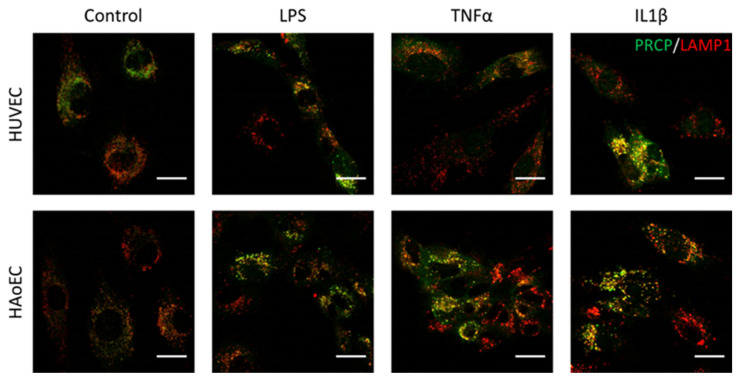
PRCP is observed in lysosomes of HUVEC and HAoEC. Double staining of PRCP and LAMP1 in control, LPS-, TNFα-, and IL-1β-stimulated HUVEC. Cells were incubated for 16 h with the different stimuli, fixed with 4% PFA, permeabilized with 0.1% Triton X-100 and stained for PRCP (green) and LAMP1 (red). Overlap of LAMP1 and PRCP was observed as yellow color. Representative images of three independent experiments. (bar = 20 µm).

**Figure 7:**
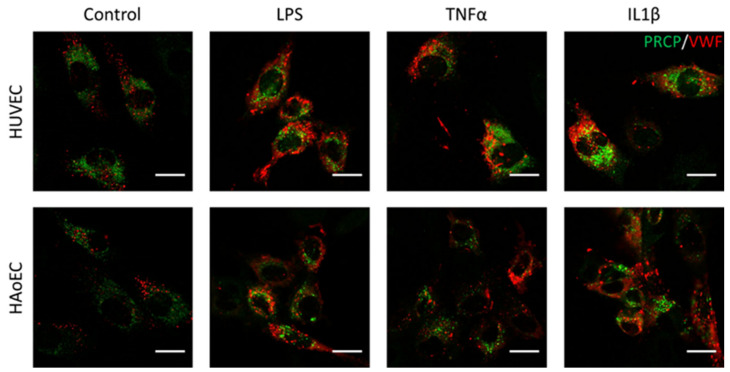
PRCP did not show overlap with vWF in stimulated HUVEC and HAoEC. Double staining of PRCP and VWF in control, LPS-, TNFα-, and IL-1β-stimulated HUVEC. Cells were incubated for 16 h with the different stimuli, fixed with 4% PFA, permeabilized with 0.1% Triton X-100 and stained for PRCP (green) and vWF (red). Representative images of two independent experiments. (bar = 20 µm).

**Figure 8:**
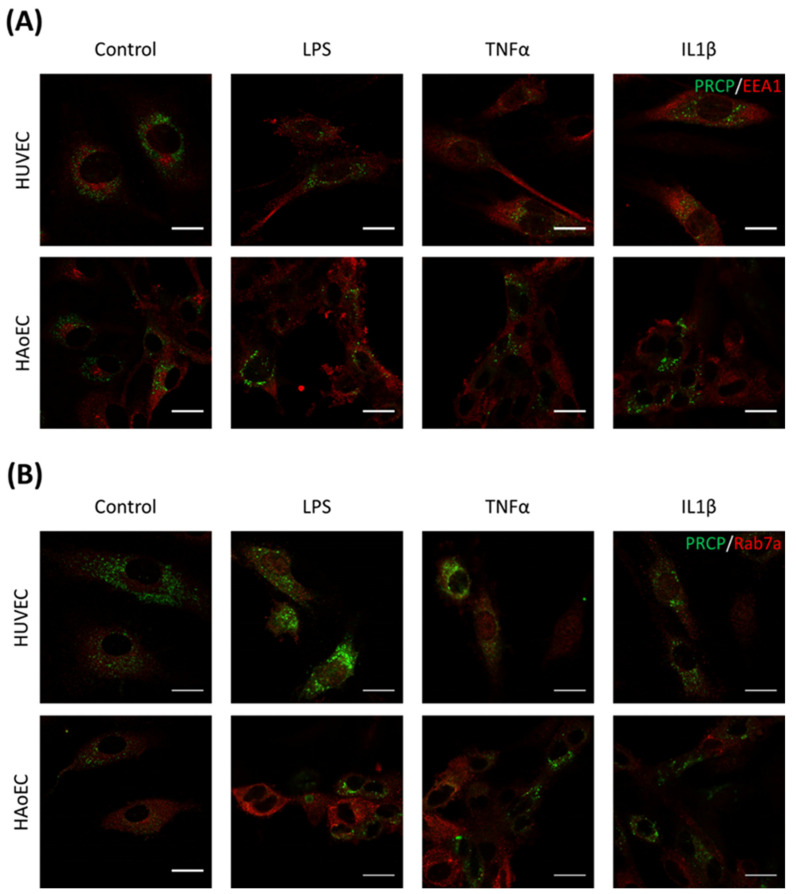
PRCP is not observed in the early or late endosomes of HUVEC or HAoEC. Double staining of PRCP and EEA1/Rab7a in control, LPS-, TNFα-, and IL-1β-stimulated HUVEC (**A**) and HAoEC (**B**). Cells were incubated for 16 h with the different stimuli, fixed with 4% PFA, permeabilized with 0.1% Triton X-100 and stained for PRCP (green) and EEA1/Rab7a (red). Representative images of three independent experiments. (bar = 20 µm).

We investigated the location of PRCP in HUVEC and HAoEC by performing double immunofluorescent staining experiments with different markers. The following markers were chosen: CD31 as endothelial cell membrane marker, LAMP1 as lysosomal marker, EEA1 as marker for the early endosomes, and Rab7a for the late endosomes and von Willebrand Factor (vWF) as endothelial cell specific marker. To examine the effect of pro-inflammatory stimuli on the location of PRCP, we used 4 conditions: control and LPS-, tumor necrosis factor (TNF)α-, and interleukin (IL)-1β-stimulated HUVEC/HAoEC. PRCP staining was observed in cytoplasmic vesicular structures in both types of endothelial cells (Figure 3). Remarkably, PRCP was not observed in all cells. The latter can be due to poor incorporation of the anti-PRCP antibody into the cells or can indicate that PRCP is not expressed in all cells. The staining of CD31 and PRCP did not show overlap in any of the conditions, in both permeabilized and non-permeabilized cells (Figure 4 and Figure 5 and Supplementary Materials File S1), indicating that PRCP is not located on the cell membrane of endothelial cells or that PRCP is only temporarily expressed on the extracellular membrane, so we could therefore not detect its cell membrane location via a confocal image on fixated cells. PRCP was found in LAMP1-positive lysosomal structures (Figure 6) in all conditions in both types of endothelial cells. Staining of PRCP did not show overlap with vWF (Figure 7) and presence of PRCP in EEA1- or Rab7a- positive endosomes could not be observed (Figure 8). All staining controls were negative (data not shown). Detailed confocal images can be found in Supplementary Materials File S1.

### 2.4. Stimulation with Pro-Inflammatory Agents Revealed an Increase in PRCP Activity in the Supernatant of HUVEC and HAoEC

**Figure 9:**
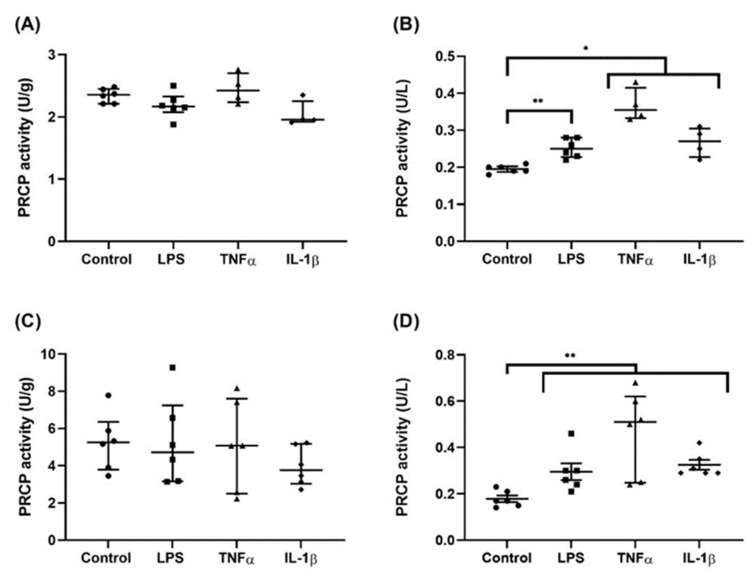
Stimulation with pro-inflammatory agents revealed an increase in PRCP activity in the supernatant of HUVEC and HAoEC. PRCP activity (U/g or U/L) in the cell lysate ((**A**) HUVEC and (**C**) HAoEC) and supernatant ((**B**) HUVEC and (**D**) HAoEC) of endothelial cells after treatment with vehicle control, LPS, TNFα or IL-1β measured with a validated RP-HPLC assay. Results are reported as Median ± IQR. (*n* = 4–6; * *p* < 0.05, ** *p* < 0.01, Kruskal-Wallis and Mann-Whitney U).

To examine the effect of pro-inflammatory stimuli on PRCP enzymatic activity, we measured PRCP activity under four conditions: control and LPS-, TNFα- and IL-1β-stimulated HUVEC/HAoEC. A significant difference in PRCP activity between the different groups was revealed in the supernatant of HUVEC (*p* = 0.001) and HAoEC (*p* = 0.003), while no difference was observed in the cell lysate (*p* = 0.066 for HUVEC and *p* = 0.563 for HAoEC). Figure 9 depicts the difference in PRCP activity in the supernatant between the control and the LPS-stimulated (*p* = 0.004 for HUVEC and *p* = 0.008 for HAoEC), TNFα-stimulated (*p* = 0.010 for HUVEC and *p* = 0.004 for HAoEC), and IL-1β-stimulated (*p* = 0.010 for HUVEC and *p* = 0.004 for HAoEC) cells. An interesting observation is the higher PRCP activity in HAoEC in comparison with HUVEC. We also measured ACE2 activity, but no difference could be found between the control and stimulated cells. It was observed that the ACE2 activity in the endothelial cell lysates was much lower than the PRCP activity. Moreover, the ACE2 activity was below the limit of quantification in the supernatant of both types of endothelial cells (see Supplementary Materials File S2).

## 3. Discussion

### 3.1. Cleavage of (Pyr)-Apelin-13 Is Attributed to PRCP and ACE2 and Differs between HUVEC and HAoEC

Historically, the C-terminal cleavage of (pyr)-apelin-13 was assigned to ACE2 [28,29,30], until we identified PRCP as an apelin-cleaving enzyme [17]. Aiming to study the role of PRCP in apelin turnover in a cellular environment, we investigated (pyr)-apelin-13 truncation in endothelial cells, including the contribution of PRCP and ACE2. We performed the experiments in HUVEC and in HAoEC, as representatives for endothelial cells of veins and arteries. The evidence shows that (pyr)-apelin-13 is cleaved at its C-terminus in HUVEC as well as in HAoEC. In HUVEC, only PRCP is responsible for this cleavage, while in HAoEC both PRCP and ACE2 are responsible.

As there is still formation of (pyr)-apelin-13_(1–12)_ after PRCP-inhibition in HUVEC or after PRCP/ACE2-inhibition in HAoEC, it is likely that also other enzymes are involved in this cleavage. As prolyl oligopeptidase (PREP, EC 3.4.21.26) cleaves after a proline, (pyr)-apelin-13 is theoretically susceptible to C-terminal cleavage by PREP, but to the best of our knowledge PREP’s putative contribution to this cleavage was never investigated previously. So, we set up a cell-independent experiment to determine whether recombinant human PREP (rhPREP) cleaves (pyr)-apelin-13 in vitro (see Supplementary Materials File S3) [48]. However, we could not observe (pyr)-apelin-13 cleavage by rhPREP, while established PREP-substrates angiotensin II and α-MSH 1–13 were cleaved under the same conditions. As (pyr)-apelin-13 is not an in vitro substrate of PREP, it is unlikely that PREP contributes significantly to the cleavage of (pyr)-apelin-13 in endothelial cells.

We also observed cleavage of (pyr)-apelin-13 between Ser6 and His7 in HAoEC, but not in HUVEC. This (pyr)-apelin-13_(1-6)_ fragment was previously identified as a metabolite of (pyr)-apelin-13 in human plasma of healthy volunteers [49]. Murza et al. also detected the cleavage site between Ser6 and His7 in rat, mouse and human plasma, but only when (pyr)-apelin-13 was first cleaved between Leu5 and Ser6, so (pyr)-apelin-13_(1-6)_ was not formed [27]. Further research into the origin of this cleavage product is needed.

### 3.2. Enzyme Activity Levels May Account For Differences in Cleavage Patterns of (Pyr)-Apelin-13 in Both Types of Endothelial Cells

It is very interesting that the enzymes involved in the conversion of (pyr)-apelin-13 and the resulting cleavage patterns differ between the two types of endothelial cells. Apart from differences in enzyme expression and/or activity, mechanisms of peptide uptake may vary between both cell types and, in certain cases, the peptide and peptidase may simply not encounter each other because they are in different cellular compartments. PRCP is a lysosomal enzyme, but it is also detected extracellularly [43]. In this study, we measured PRCP enzymatic activity both inside the cell and in the extracellular environment. Pro-inflammatory stimulation of endothelial cells increased the secretion of PRCP in the cell medium, while its intracellular level remained intact. Furthermore, PRCP activity was higher in HAoEC than in HUVEC. ACE2 is a type I transmembrane protein with an extracellular domain containing the active site and also has an active soluble form [50]. We were not able to detect an active form of ACE2 in the cell culture medium of endothelial cells and the activity in the cell lysate was very low. The ACE2 activity in HAoEC was twice as high as in HUVEC and this may account for the difference in ACE2-dependent cleavage between both types of endothelial cells.

### 3.3. PRCP’s Location in Endothelial Cells Can Be Related to Its Role in the Cleavage of Circulating Peptides

As we could only detect the cleaved apelin forms in the cell supernatant after a longer incubation time of 8 h or 24 h, we hypothesized that the peptide is taken up into the cell, processed intracellularly, and secreted subsequently. To verify whether secreted PRCP is responsible for this cleavage, we incubated the 24 h cellular supernatant of HUVEC with (pyr)-apelin-13 in the absence of cells during 1 h (Supplementary Materials File S4). If secreted PRCP is responsible for the C-terminal cleavage of (pyr)-apelin-13, we should observe the formation of significant levels of (pyr)-apelin-13_(1–12)_ in this experiment. Although some PRCP-mediated cleavage of (pyr)-apelin-13 occurred, this was to a much lesser extent than in the original cellular experiment. Therefore, secreted PRCP is most likely not a major player in the C-terminal cleavage of (pyr)-apelin-13, supporting our hypothesis on intracellular processing.

Knowledge about PRCP’s location in and on these cells is imperative for a better understanding of our hypothesis. Therefore, we investigated PRCP’s location with immunocytochemistry experiments. Previously, Shariat-Madar et al. showed that PRCP is constitutively expressed on the membrane of cultured endothelial cells by staining experiments on non-permeabilized HUVEC and co-localization experiments with receptors for high-molecular weight kininogen [3,40]. However, these results were not confirmed with double staining experiments with the established endothelial cell membrane marker CD31. Furthermore, the authors observed only partial co-localization between PRCP and LAMP1 in HUVEC, confirming the location of PRCP in lysosomes, but also suggesting that PRCP is expressed in other organelles [40]. The latter, and the fact that PRCP is found extracellularly as soluble protein in urine, plasma, and synovial fluid and as part of the secretome of human endothelial cells [43,44], can indicate that PRCP is transported via the endosomal pathway and secreted in the extracellular environment. So, we hypothesized that (pyr)-apelin-13 could be processed intracellularly by PRCP and the cleavage product is secreted via the endosomal pathway, if peptide and enzyme are both trafficked via this pathway.

We did confirm the presence of PRCP in lysosomes but could not observe presence of PRCP in the other studied organelles, namely Weibel-Palade Bodies and endosomes. Overlap with vWF could not be detected. As vWF is stored in Weibel-Palade Bodies, which are lysosome-related secretory vesicles of endothelial cells which also store P-selectin, PRCP is probably not present in these organelles. As we could not observe overlap between PRCP and endosomal markers EEA1 and Rab7a, our hypothesis that PRCP is trafficked via the endosomal pathway is not supported. We assume that PRCP-mediated apelin cleavage takes places in the lysosomes, which are a perfect environment for PRCP-mediated processing given the acidic pH optimum of PRCP. We hypothesize that apelin is trafficked via the endo-lysosomal pathway into lysosomes where it is truncated by PRCP. The cleaved product is then secreted via lysosomal exocytosis [51,52,53]. Double staining experiments of PRCP and apelin should give more clarity about this hypothesis in the future. Furthermore, it must be recognized that these organelles form a continuum rather than a defined organelle group and so it is challenging to differentiate late endosomes from lysosomes based on these markers [53].

In the past, PRCP was identified as the prekallikrein activator (PKA) on the cell membrane of HUVEC [3]. We could not confirm cell membrane location of PRCP in endothelial cells, although the CD31 staining was clearly membranous. At first sight, these results seemed surprising. However, it has been shown by Shariat-Madar et al. that PKA activity was mainly associated with the granule-lysosomal fraction of HUVEC and PRCP was enriched from this fraction. It was suggested that a part of the lysosomal pool is expressed on the external membrane of endothelial cells [3]. These data, together with the present study, support the hypothesis that PRCP is constitutively expressed in endothelial lysosomes and that PRCP, derived from endothelial lysosomes, might be temporarily expressed on the extracellular membrane during prekallikrein activation. However, we do not have direct evidence for this. The discrepancies on PRCP cell membrane location might also be caused by the use of different antibodies that recognize distinct epitopes.

### 3.4. The Studied Enzymes Do Not Seem to Be Involved to a Significant Extent in the Cleavage of α-MSH 1–13 in Human Endothelial Cells

For comparison, we also tested the cleavage of another PRCP-substrate, α-MSH 1–13 [10]. This anorexigenic peptide is cleaved at its C-terminus in function of time and only the combined inhibition of PRCP and ACE2 slightly reduced the formation of α-MSH 1–12. Thus, none of the studied enzymes seem to be involved to a significant extent in the C-terminal cleavage of α-MSH 1–13 in endothelial cells. PRCP is described as an MSH-cleaving enzyme [10,11], so these results are rather surprising. Firstly, one possibility is that PRCP and α-MSH 1–13 may not encounter each other because they are in different cellular compartments and therefore the cleavage cannot occur. Secondly, it is conceivable that the ideal circumstances (e.g., pH) for cleavage are not met. The formation of α-MSH 1–12 seen in function of time is probably caused by other enzymes. As α-MSH 1–13 is a reported substrate of PREP [54], we also tested the contribution of this enzyme in endothelial cell culture experiments by use of the PREP-inhibitor KYP-2047 [55]. However, these experiments were inconclusive and therefore not included. PREP is involved in the regulation of autophagy and KYP-2047 acts as an autophagy inducer [56], thus indirectly affecting peptide and protein degradation in general. These cellular effects of PREP-inhibition can influence our results and offer the perspective to study PREP during autophagy induction in endothelial cells.

### 3.5. Limitations

A limitation of the current study is that a cell culture environment may not reflect an in vivo setting. Moreover, we exogenously add the peptides to the cells, as their endogenous concentrations are too low to detect or dissect their cleavage patterns. We have tried to silence PRCP and ACE2 in endothelial cells in order to confirm the results obtained with our pharmacological approach using selective enzyme inhibitors. However, we did not succeed to produce PRCP- and ACE2-silenced cells in a reproducible manner. Although these experiments might not reflect the exact in vivo situation, our findings contribute to a better understanding of (pyr)-apelin-13 cleavage in endothelial cells. For the first time, the cleavage of (pyr)-apelin-13 was investigated in a cellular environment in the presence or absence of specific inhibitors for PRCP and ACE2.

## 4. Materials and Methods

### 4.1. Inhibitors

The PRCP-inhibitor compound 8o [46] was custom synthesized in the Department of Medicinal Chemistry of the Latvian Institute of Organic Synthesis. The ACE2-inhibitor DX600 [47] was purchased from BioVision (Milpitas, CA, USA).

### 4.2. Substrates

(Pyr)-apelin-13 (pERPRLSHKGPMPF), α-MSH 1–13 (Ac-SYSMEHFRWGKPV-NH₂), and N-benzyloxycarbonyl-Pro-Phe (Z-Pro-Phe) were purchased from Bachem (Bubendorf, Switzerland).

### 4.3. Cell Culture

HUVEC and HAoEC were obtained from PromoCell (Heidelberg, Germany) and Cell Applications (San Diego, CA, USA), respectively, and cultured in endothelial cell growth medium (R&D systems, Minneapolis, MN, USA) supplemented with antibiotics (100 U/mL penicillin and 100 µg/mL streptomycin; Gibco, Waltham, MA, USA) at 37 °C in 5% CO_2_. Medium was replaced every 2–3 days. After reaching 70–80% confluence, HUVEC and HAoEC were detached using TrypLE^TM^ Express (Life Technologies, Carlsbad, CA, USA). Cells were counted using the Scepter^TM^ 2.0 Cell Counter with a 60 µM Scepter sensor (Merck-Millipore, Burlington, MA, USA). Passage numbers 2–8 were used for HUVEC and 2–5 for HAoEC.

### 4.4. Substrate Cleavage in Endothelial Cells

HUVEC and HAoEC were seeded at a density of 10,000 cells per well in 96-well plates in 100 µL full medium. After 24 h, cells were treated with vehicle control (1% DMSO), 1 µM compound 8o, 1 µM DX600, or the two inhibitors (all final concentration of 1% DMSO) in assay medium (5% full medium in Hank’s Balanced Salt Solution (HBSS)) for 15 min at 37 °C in 5% CO_2_. This resulted in four treatment options (control, PRCP-inhibited, ACE2-inhibited and PRCP/ACE2-inhibited cells). Then, 100 μM substrate ((pyr)-apelin-13 or α-MSH 1–13) or vehicle control (PBS) was added to the wells. The reaction was stopped by acidification (pH < 3) with 0.1% trifluoroacetic acid (TFA) after five different time periods (0 h, 2 h, 4 h, 8 h or 24 h) and supernatants were stored at −80 °C until further processing. The experiment was independently conducted 4 times. Before the start of the experiments, viability of HUVEC and HAoEC in the presence of compound 8o or DX600 was assessed with PrestoBlue^®^ Cell Viability Reagent (Life Technologies, Carlsbad, CA, USA) and inhibitor potency was analyzed (Supplementary Materials Files S5 and S6) [57].

### 4.5. MALDI-TOF/TOF Analysis

To detect the cleavage of (pyr)-apelin-13 (*m/z* 1533.8) and α-MSH 1–13 (*m/z* 1665.8), the samples were analyzed by matrix-assisted laser desorption/ionization time-of-flight/TOF (MALDI-TOF/TOF). The supernatants were desalted and concentrated by use of C_18_ ZipTips (Merck-Millipore, Burlington, MA, USA) and eluted directly on the MALDI target in 70% acetonitrile/0.1% TFA. Subsequently, a MALDI matrix solution of 2.5 mg/mL α-cyano-4-hydroxycinnamic acid in 70% acetonitrile/0.1% TFA was applied on each spot, air-dried at room temperature, and the target was introduced into a 4800 plus MALDI TOF/TOF^TM^ Analyzer. Mass spectrometry (MS) spectra (mass range: 600–4000 Da) were acquired in positive reflector mode with a laser intensity of 5000. To confirm the identity of the most important peptides, MS/MS spectra were generated by use of collision induced dissociation with a laser intensity of 5500. Calibration was done during analysis using the 6-peptide mixture (AB Sciex, Framingham, MA, USA). Mass spectra were generated by use of the 4000 Series Explorer and analyzed by mMas [58]. Hydrolysis of the substrates at the C-terminus would lead to additional product peaks in the mass spectrum of respectively (pyr)-apelin-13_(1–12)_ (*m/z* 1386.7) and α-MSH 1–12 (*m/z* 1566.7). The ratio of the peak intensity of the cleaved substrate to the peak intensity of the intact substrate is an indication for the C-terminal cleavage of the substrates. To check for additional cleavage sites, a search for other product peaks was completed. Statistical analysis was performed using SPSS software version 27 (IBM). Two-way ANOVA analysis was conducted to examine the effect of the two independent variables (treatment duration and treatment option) on the ratio of the peak intensities. For results that showed a significant interaction between the effects of treatment duration and treatment option on the ratio of the peak intensities, simple main effects analysis was executed. When there was no significant interaction, main effects were interpreted, and Tukey Post hoc analysis was conducted for the variables that showed significant main effects. *p* < 0.05 was considered as statistical significance and significant results are indicated on the graphs (GraphPad Prism 9).

### 4.6. Immunocytochemistry

HUVEC and HAoEC were seeded in Nunc™ Lab-Tek™ II CC2™ Chamber Slides at a density of 21,000 cells per well and incubated for 24 h at 37 °C. The next day, cells were treated with 200 ng/mL LPS (Immunotools, Friesoythe, Germany), 10 ng/mL TNFα (Immunotools, Friesoythe, Germany), 5 ng/mL IL-1β (Immunotools, Friesoythe, Germany), or vehicle control (PBS) for 16 h. Subsequently, the cells were washed with HBSS and fixed with 4% paraformaldehyde (PFA) for 30 min at room temperature. After a washing step with PBS the cells were permeabilized with 0.1% Triton X-100 in blocking buffer (2% bovine serum albumin, 5% normal horse serum in PBS) for 10 min, followed by a subsequent blocking step in blocking buffer without Triton X-100 for 20 min at room temperature. Next, the cells were incubated overnight at 4 °C with primary antibodies against PRCP (HPA017065, Sigma-Aldrich, St. Louis, MO, USA, 1:50) and the other markers (one marker/slide): CD31 (Abcam (Cambridge, UK), ab24590, 1:2000), LAMP1 (Abcam (Cambridge, UK), ab25630, 1:100), EEA1 (Novus Biologicals (Centennial, CO, USA), H00008411, 1:250), Rab7a (Novus Biological (Centennial, CO, USA), NBP2-60237, 1:100), and vWF (Invitrogen (Carlsbad, CA, USA), MA5-14029, 1:100), all diluted in blocking buffer. All primary antibodies have been used in peer-reviewed articles before, references are mentioned at the suppliers’ websites. After washing in PBS, the cells were incubated with secondary antibodies for 1 h at room temperature protected from light. The secondary antibody FITC goat anti-rabbit IgG (BD Biosciences (San Jose, CA, USA), 554020) was used to visualize the primary antibody against PRCP. Alexa Fluor 594 goat anti-mouse IgG (ThermoFisher Scientific (Waltham, MA, USA), A-11005) was used to visualize the other primary antibodies. The secondary antibodies were diluted 1:200 in blocking buffer. The slides were covered with a cover glass using Vectashield antifade mounting medium with DAPI (Vector Laboratories, Peterborough, UK). For the CD31 staining, two conditions were performed: one with permeabilized cells and one with non-permeabilized cells. The slides were visualized on the inverted Leica TCS SP8 confocal laser scanning microscope. The staining was checked for autofluorescence and non-specific binding of the secondary antibodies. Normal rabbit IgG (Invitrogen, Carlsbad, CA, USA) and normal mouse IgG (Dako, Santa Clara, CA, USA) were used as isotype control. Images are all represented as a single confocal plane or single optical section from a complete z-stack through the cells.

### 4.7. Stimulation of HUVEC and HAoEC with Pro-Inflammatory Stimuli LPS, TNFα or IL-1β

HUVEC and HAoEC were seeded in a 6-well plate at a density of 2.5 × 10^5^ cells per well in 2 mL full medium for 24 h. Subsequently, cells were treated with 200 ng/mL LPS, 10 ng/mL TNFα, 5 ng/mL IL-1β, or vehicle control for 16 h. Subsequently, supernatants were collected and concentrated four times for PRCP activity measurements using Centriprep^®^ Centrifugal Filter Devices, 30 K (Merck-Millipore, Burlington, MA, USA). For PRCP activity measurement, cells were collected per well and lysed 1 h on ice in 75 µL lysis buffer (1% octylglucoside, 10 mM EDTA, 70 µg/mL aprotinin, 50 mM Tris pH 8.3). Appropriate stimulation was confirmed by determining interleukin-6 (IL-6) and interleukin-8 (IL-8) levels in the 16 h aspirate by use of ELISA (Supplementary Materials File S7) [59,60].

### 4.8. PRCP Activity Measurement

PRCP activity was measured in the cellular supernatant and cell lysate using a validated high-performance liquid chromatography (HPLC) method as described previously [61]. Ten µL of each sample was incubated in duplicate with 75 µL 8 mM Z-Pro-Phe at pH 5 (0.1 M Natrium Acetate, 10 mM EDTA) for 2 h at 37 °C. To stop the enzymatic reaction, 75 µL stop solution (10% perchloric acid and 20% acetonitrile in purified water) was added and samples were centrifuged at 12,000× *g* for 10 min at 4 °C. Ten µL of the supernatant was injected into a reversed phase HPLC system (Shimadzu, Kioto, Japan) and the enzymatically formed Z-Pro was determined by its UV absorbance at 210 nm. Quantification was performed by peak height measurements. The PRCP activity is expressed as units per gram (U/g) protein for the cell lysate or units per liter (U/L) for the supernatant. One unit defines the amount of enzyme that hydrolyses 1 μmol of substrate per minute. Protein concentrations were determined via the Bradford method with bovine serum albumin as the standard protein. Statistical analyses were performed using SPSS software version 27 (IBM) and graphs were designed using Graphpad Prism 9 software. Differences in PRCP activity in the cell lysate and supernatant between control and stimulated cells were evaluated by Kruskal-Wallis and Mann-Whitney U-tests. Manual Bonferroni-correction was assessed for multiple testing based on relevant pairwise comparisons. Significant results are indicated with an asterisk (* *p* < 0.05, ** *p* < 0.01).

## 5. Conclusions

In conclusion, we demonstrate here that (pyr)-apelin-13 is cleaved at its C-terminus in HUVEC and HAoEC. The enzymes involved differ between the two cell types. In HUVEC, the cleavage is mediated only by PRCP, while in HAoEC, also ACE2 contributes to this cleavage. This information can be of great value to further elucidate the role of the C-terminal Phe of (pyr)-apelin-13 in the future. Moreover, we confirm that PRCP is found in endothelial lysosomes. Pro-inflammatory stimulation induces PRCP secretion, while its activity level inside the cells remains constant.

## Data Availability

The data presented in this study are available on request from the corresponding author.

## Supplementary Materials

The following are available online at https://www.mdpi.com/article/10.3390/ijms22136698/s1.
