# Supplementary material for: Prolyl Carboxypeptidase Mediates the C-Terminal Cleavage of (Pyr)-Apelin-13 in Human Umbilical Vein and Aortic Endothelial Cells
Source: Int J Mol Sci. 2021 Jun 22;22(13):6698. doi: 10.3390/ijms22136698 (PMC8268575; doi:10.3390/ijms22136698)
Supplement: Supplementary file 1 [file ijms-22-06698-s001.zip › Supplementary Material File 1.pdf]

Supplementary Material File S1. Confocal images

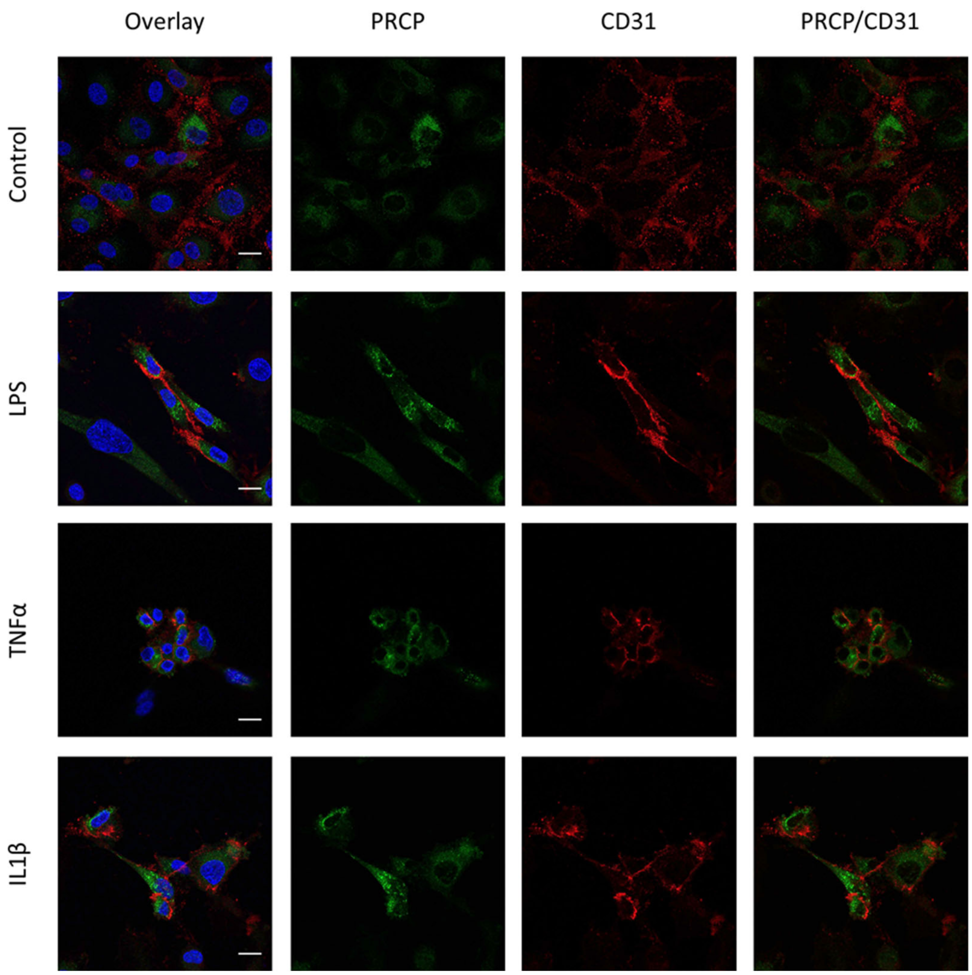

Figure S1.1: Double staining of PRCP and CD31 in control, LPS-, TNFα- and IL-1β-stimulated permeabilised HUVEC. Cells were incubated for 16 h with the different stimuli, fixed with 4% PFA, permeabilised with 0.1% Triton X-100 and stained for PRCP (green), CD31 (red) and DAPI (nuclear marker, blue). Representative images of three independent experiments (bar = 20 μm). Non cropped figures.

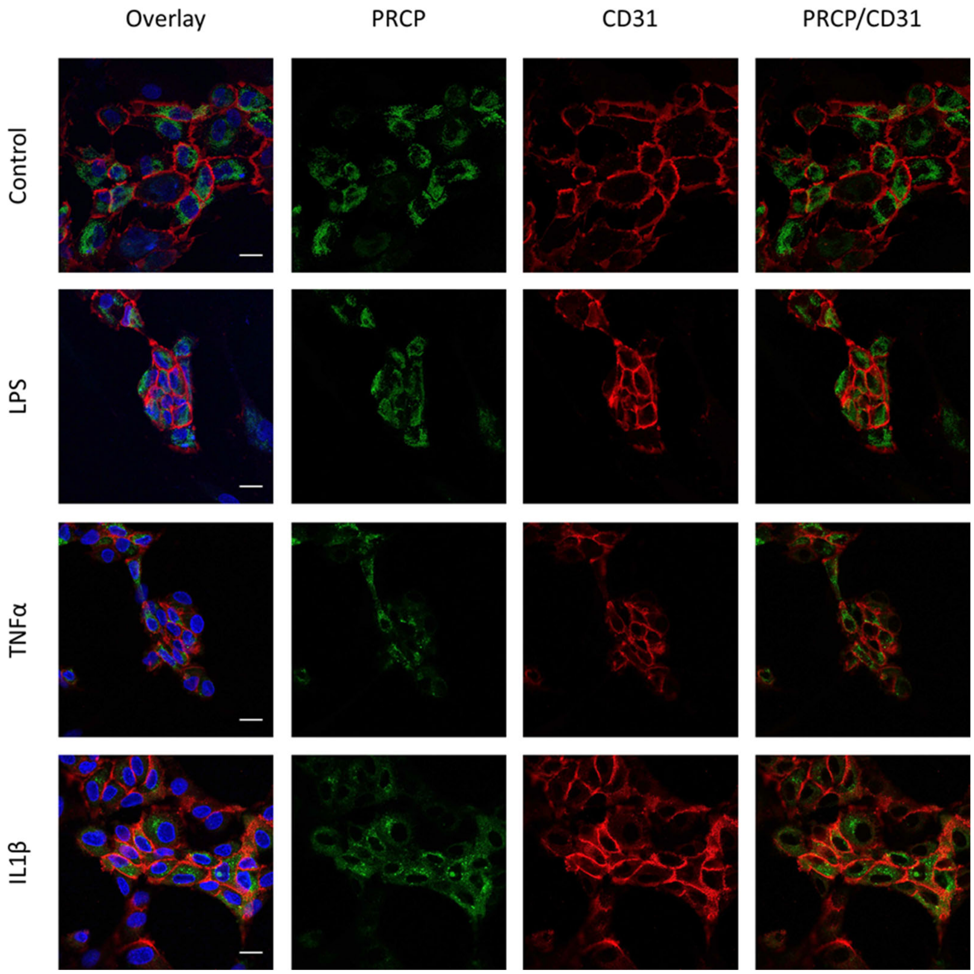

Figure S1.2: Double staining of PRCP and CD31 in control, LPS-, TNFα- and IL-1β-stimulated permeabilised HAoEC. Cells were incubated for 16 h with the different stimuli, fixed with 4% PFA, permeabilised with 0.1% Triton X-100 and stained for PRCP (green), CD31 (red) and DAPI (nuclear marker, blue). Representative images of three independent experiments (bar = 20 µm). Non cropped figures.

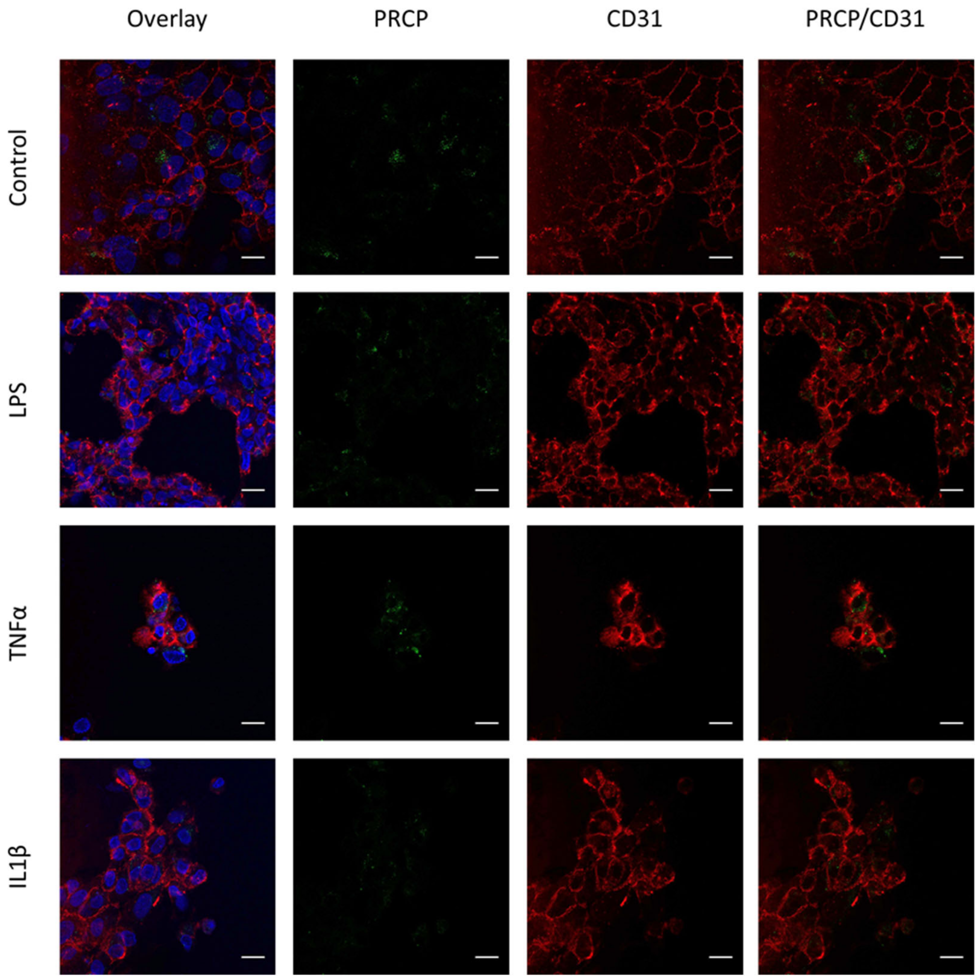

Figure S1.3: Double staining of PRCP and CD31 in control, LPS-, TNFα- and IL-1β-stimulated non-permeabilised HUVEC. Cells were incubated for 16 h with the different stimuli, fixed with 4% PFA and stained for PRCP (green), CD31 (red) and DAPI (nuclear marker, blue). Representative images of three independent experiments. (bar = 20 µm)

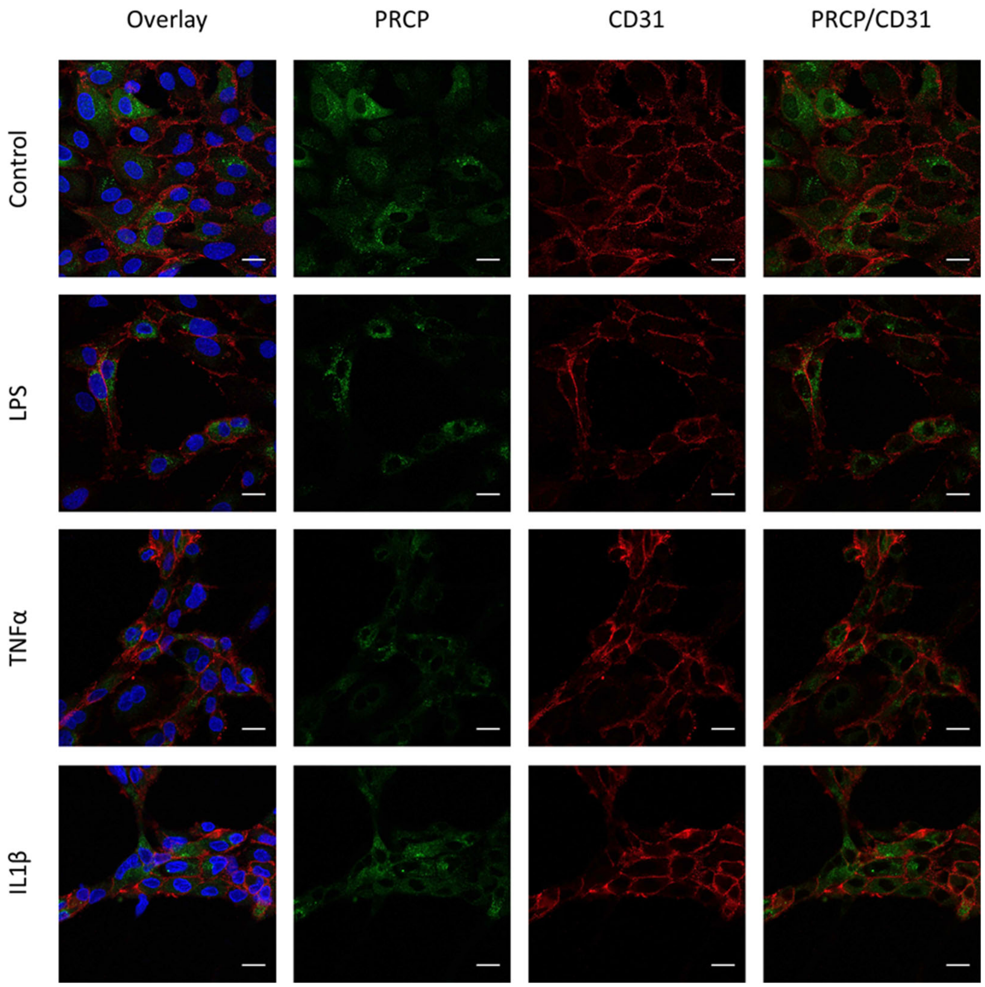

Figure S1.4: Double staining of PRCP and CD31 in control, LPS-, TNFα- and IL-1β-stimulated non-permeabilised HAoEC. Cells were incubated for 16 h with the different stimuli, fixed with 4% PFA and stained for PRCP (green), CD31 (red) and DAPI (nuclear marker, blue). Representative images of three independent experiments. (bar = 20 μm)

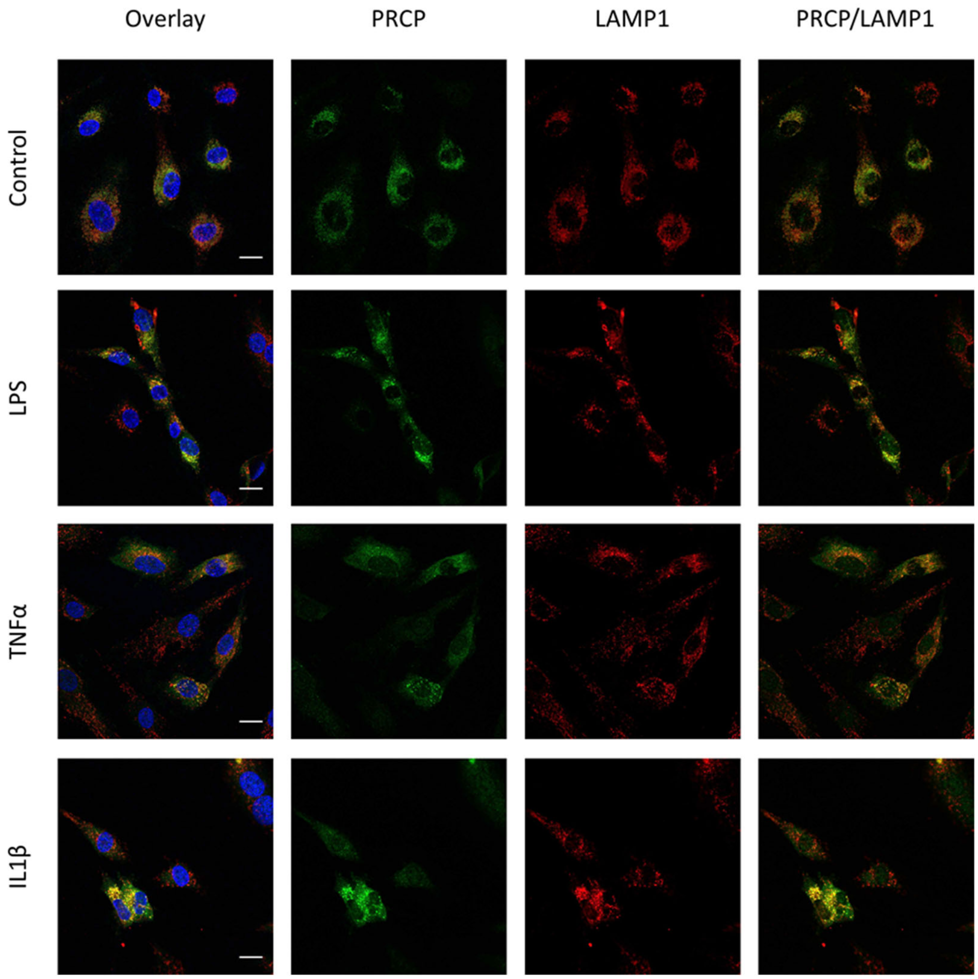

Figure S1.5: Double staining of PRCP and LAMP1 in control, LPS-, TNFα- and IL-1β-stimulated HUVEC. Cells were incubated for 16 h with the different stimuli, fixed with 4% PFA, permeabilised with 0.1% Triton X-100 and stained for PRCP (green), LAMP1 (red) and DAPI (nuclear marker, blue). Overlap of LAMP1 and PRCP was observed as yellow colour. Representative images of three independent experiments. (bar = 20 µm)

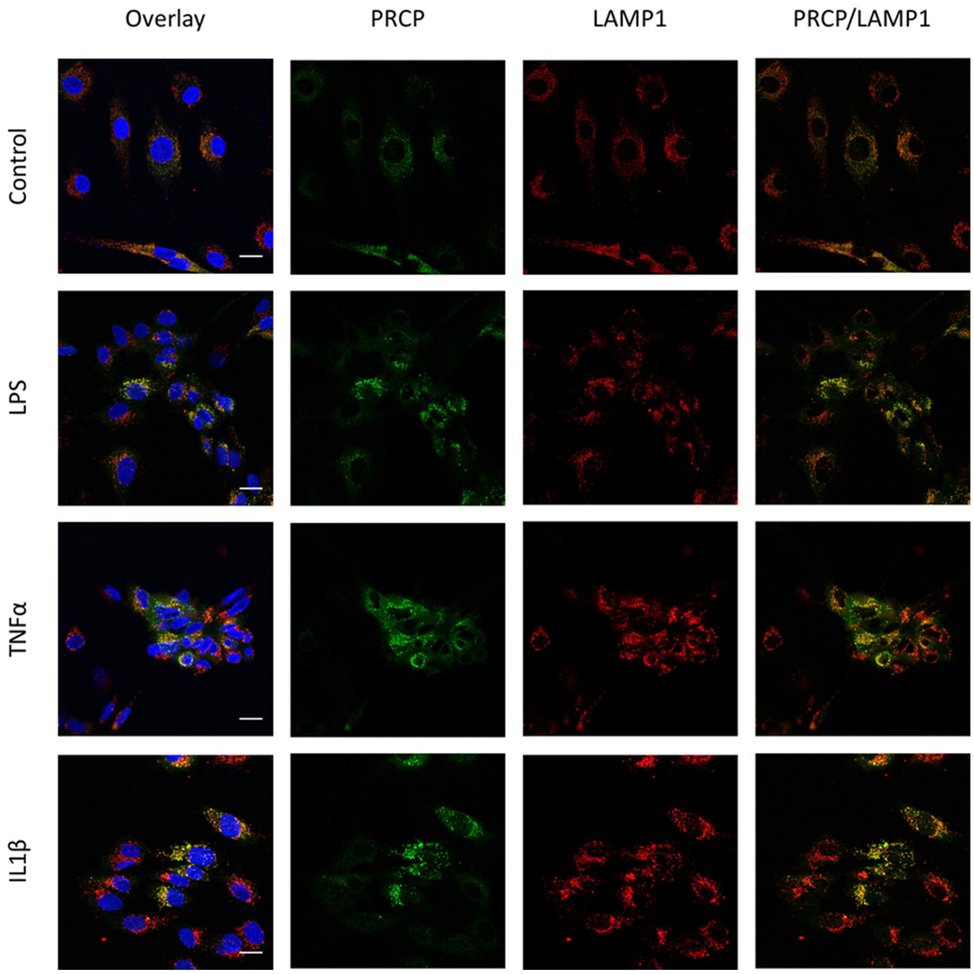

Figure S1.6: Double staining of PRCP and LAMP1 in control, LPS-, TNFα- and IL-1β-stimulated HAoEC. Cells were incubated for 16 h with the different stimuli, fixed with 4% PFA, permeabilised with 0.1% Triton X-100 and stained for PRCP (green), LAMP1 (red) and DAPI (nuclear marker, blue). Overlap of LAMP1 and PRCP was observed as yellow colour. Representative images of three independent experiments. (bar = 20 µm)

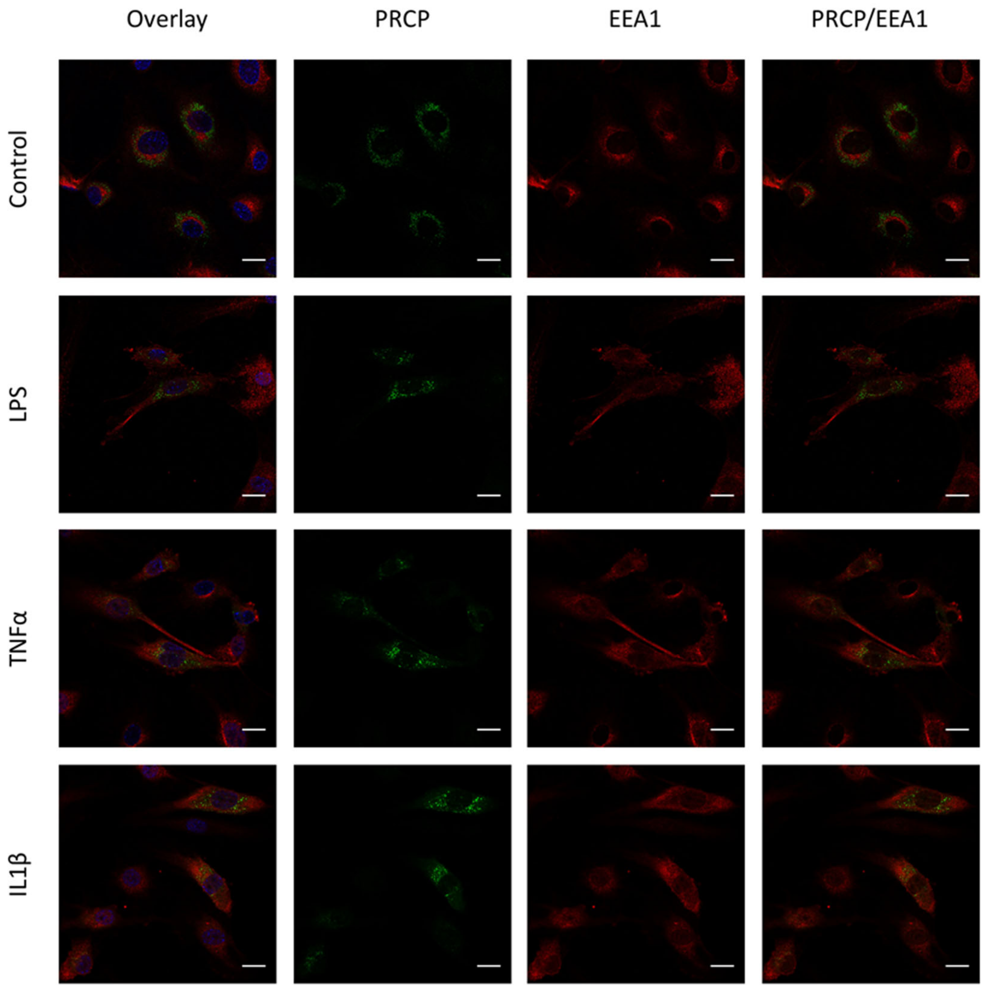

Figure S1.7: Double staining of PRCP and EEA1 in control, LPS-, TNFα- and IL-1β-stimulated HUVEC. Cells were incubated for 16 h with the different stimuli, fixed with 4% PFA, permeabilised with 0.1% Triton X-100 and stained for PRCP (green), EEA1 (red) and DAPI (nuclear marker, blue). Representative images of three independent experiments. (bar = 20 μm)

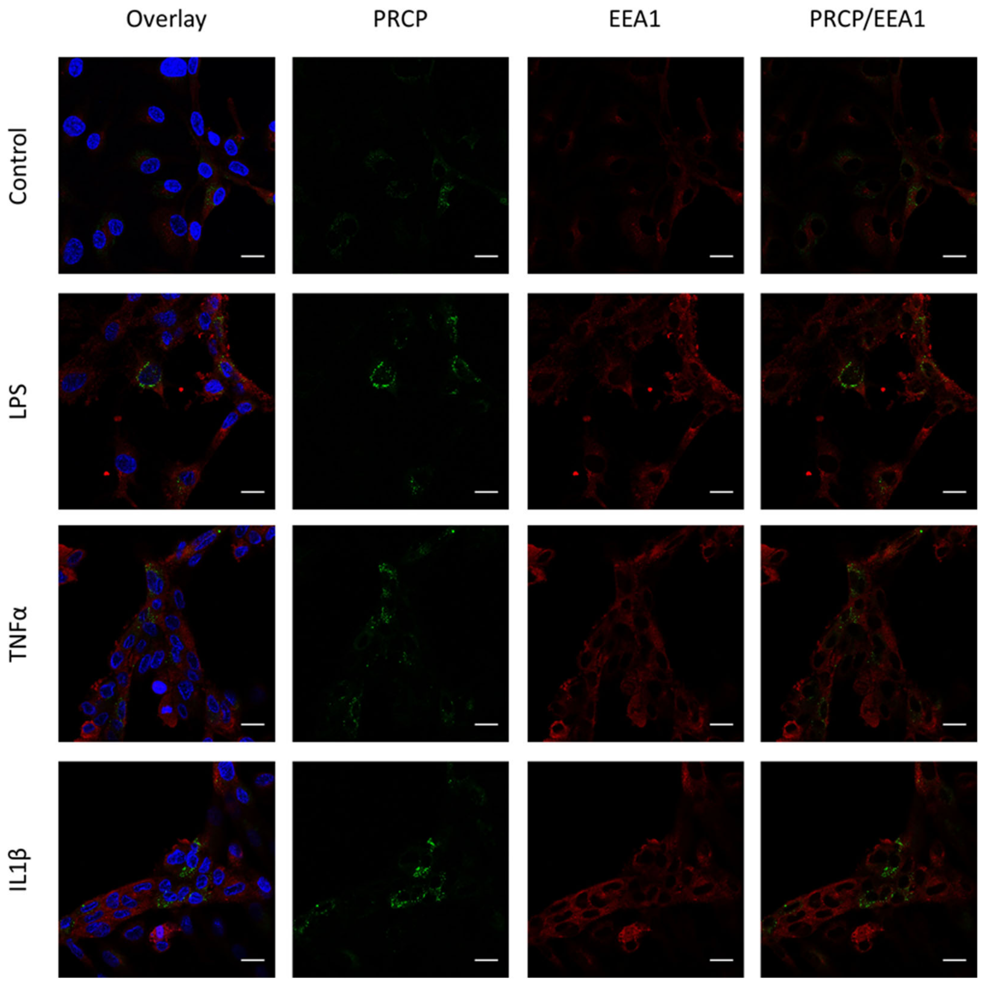

Figure S1.8: Double staining of PRCP and EEA1 in control, LPS-, TNFα- and IL-1β-stimulated HAoEC. Cells were incubated for 16 h with the different stimuli, fixed with 4% PFA, permeabilised with 0.1% Triton X-100 and stained for PRCP (green), EEA1 (red) and DAPI (nuclear marker, blue). Representative images of three independent experiments. (bar = 20 µm)

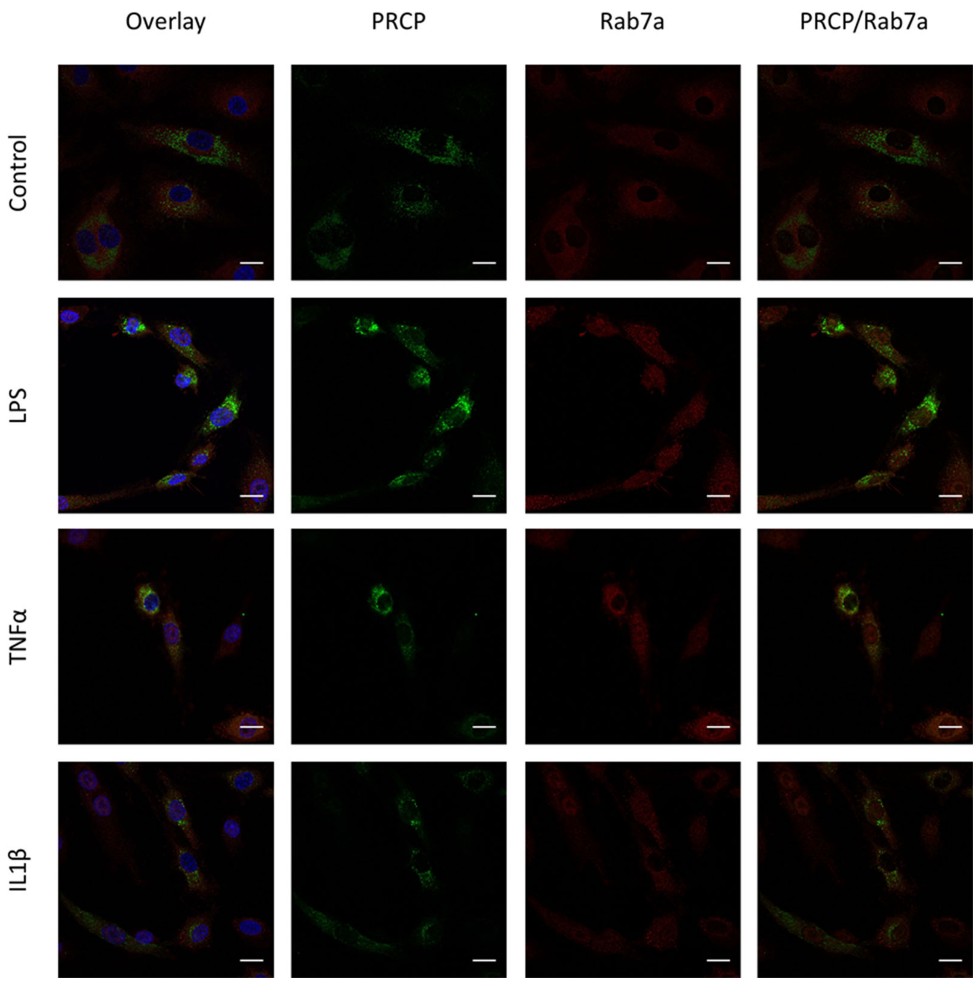

Figure S1.9: Double staining of PRCP and Rab7a in control, LPS-, TNFα- and IL-1β-stimulated HUVEC. Cells were incubated for 16 h with the different stimuli, fixed with 4% PFA, permeabilised with 0.1% Triton X-100 and stained for PRCP (green), Rab7a (red) and DAPI (nuclear marker, blue). Representative images of three independent experiments. (bar = 20 μm)

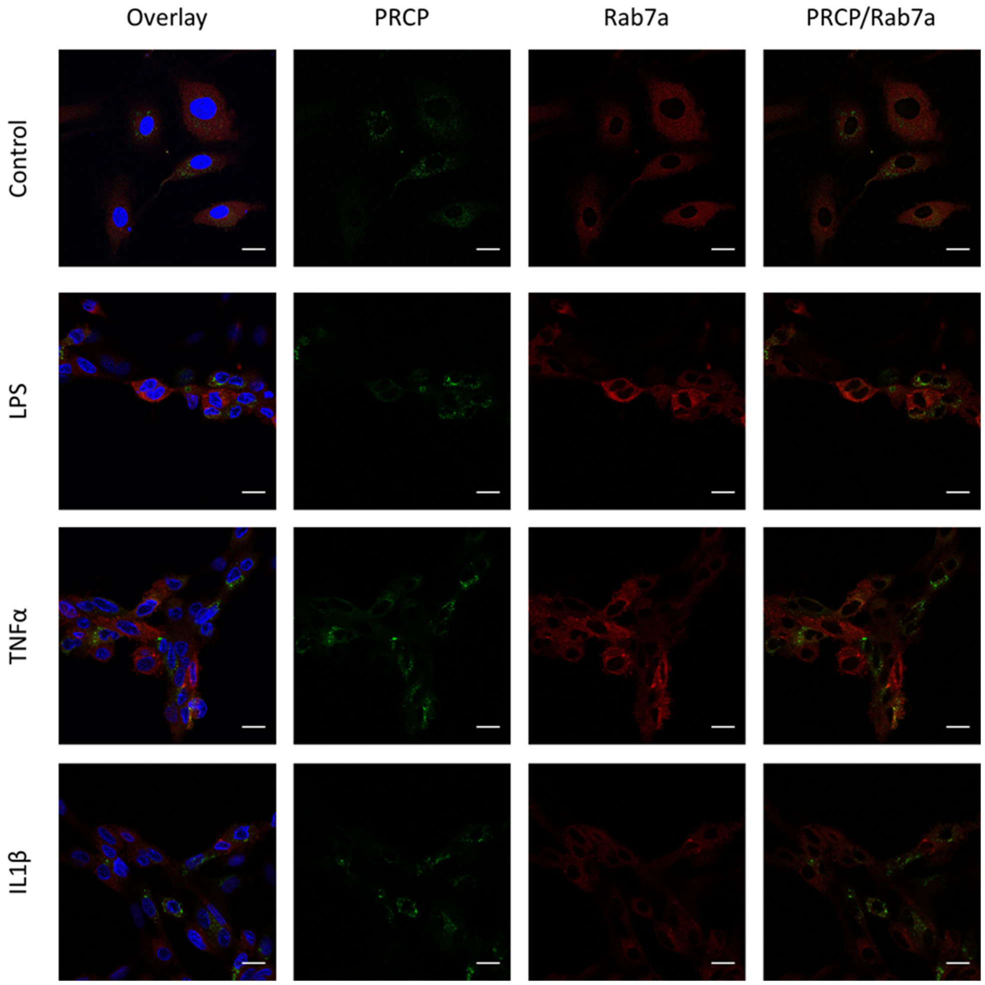

Figure S1.10: Double staining of PRCP and Rab7a in control, LPS-, TNFα- and IL-1β-stimulated HAoEC. Cells were incubated for 16 h with the different stimuli, fixed with 4% PFA, permeabilised with 0.1% Triton X-100 and stained for PRCP (green), Rab7a (red) and DAPI (nuclear marker, blue). Representative images of three independent experiments. (bar = 20 µm)

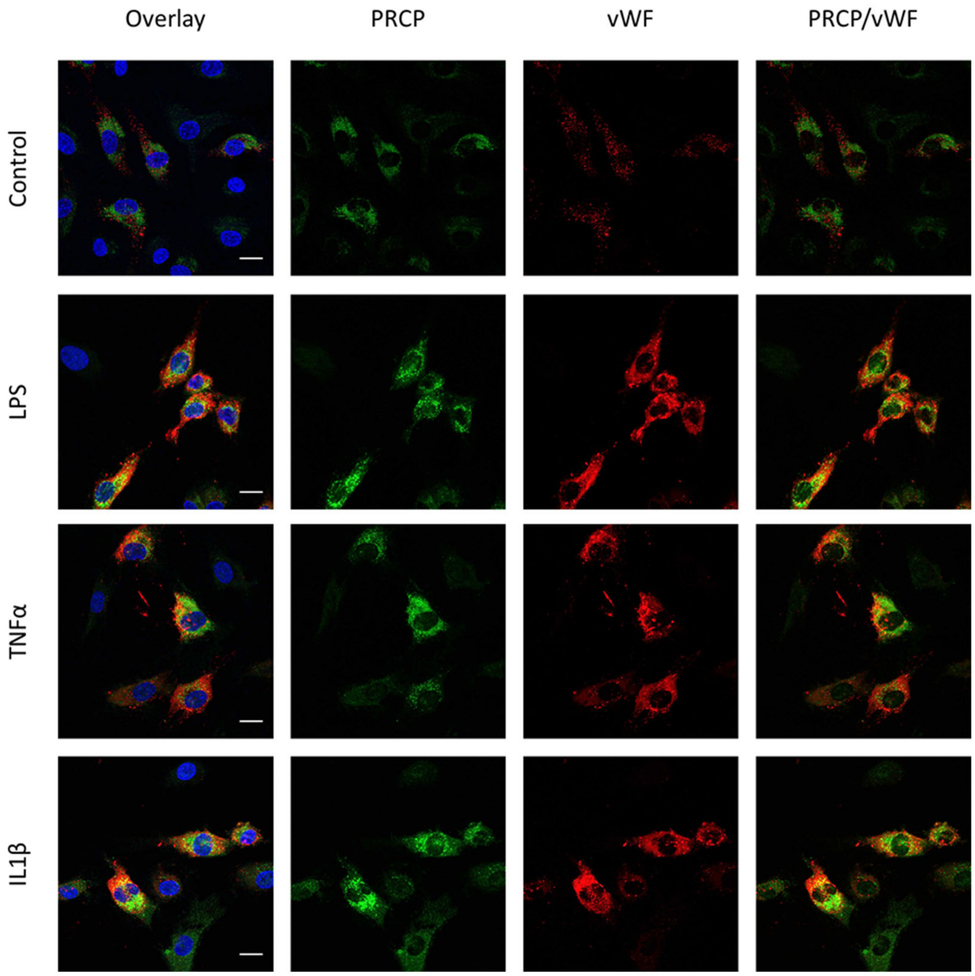

Figure S1.11: Double staining of PRCP and VWF in control, LPS-, TNFα- and IL-1β-stimulated HUVEC. Cells were incubated for 16 h with the different stimuli, fixed with 4% PFA, permeabilised with 0.1% Triton X-100 and stained for PRCP (green), VWF (red) and DAPI (nuclear marker, blue). Representative images of three independent experiments. (bar = 20 μm)

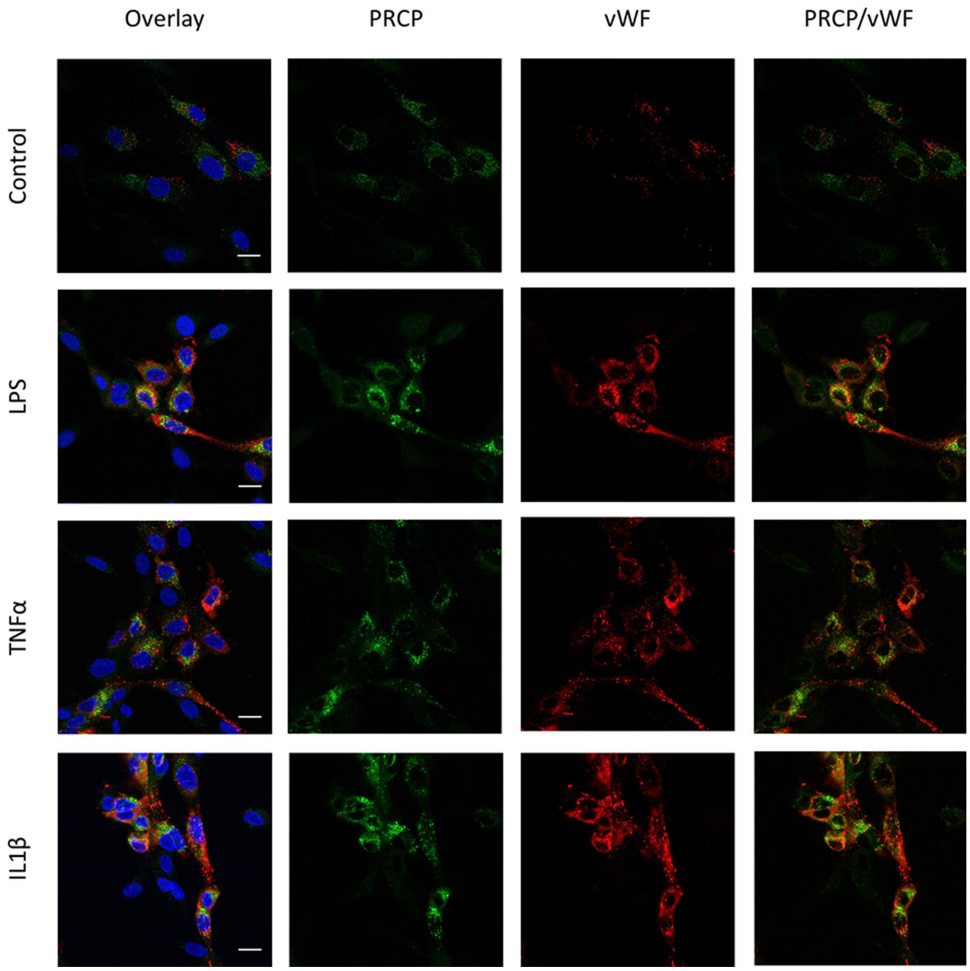

Figure S1.12: Double staining of PRCP and VWF in control, LPS-, TNFα- and IL-1β-stimulated HAoEC. Cells were incubated for 16 h with the different stimuli, fixed with 4% PFA, permeabilised with 0.1% Triton X-100 and stained for PRCP (green), VWF (red) and DAPI (nuclear marker, blue). Representative images of three independent experiments. (bar = 20 μm)
