# Supplementary material for: Prolyl Carboxypeptidase Mediates the C-Terminal Cleavage of (Pyr)-Apelin-13 in Human Umbilical Vein and Aortic Endothelial Cells
Source: Int J Mol Sci. 2021 Jun 22;22(13):6698. doi: 10.3390/ijms22136698 (PMC8268575; doi:10.3390/ijms22136698)
Supplement: Supplementary file 1 [file ijms-22-06698-s001.zip › Supplementary Material File 2.pdf]

**Supplementary Material File S2. ACE2 activity measurement**

HUVEC and HAoEC were seeded in a 6-well plate at a density of $2.5x10^5$ cells per well in 2 mL full medium for 24 h. Cells were treated with 200 ng/mL LPS, 10 ng/mL TNFα, 5 ng/mL IL-1β or vehicle control for 16 h. Supernatants were collected and concentrated four times using Centriprep Centrifugal Filter Devices, 30 K (Merck-Millipore). Cells were collected per well and lysed 1 h on ice in 75 µL lysis buffer (1% octyl glucoside, 10 mM EDTA, 70 µg/mL aprotinin, 50 mM Tris pH 8.3).

ACE2 activity was measured using the substrate Mca-Ala-Pro-Lys(Dnp)-OH (Bachem). Samples were incubated with 100 µM of the substrate at pH 6.5 (50 mM MES, 300 mM NaCl, 10 µM $ZnCl_2$, 0.01% Triton X-100) in a black 96-well plate and ACE2 activity was measured kinetically for 1 h at 37 °C ($\lambda_{ex}$=320 nm, $\lambda_{em}$=405 nm), using the Infinite 200 microplate reader (Tecan) and the Magellan software was used to process the data. Concentration of the generated Mca-Ala-Pro was determined by means of a standard curve. Specificity was determined by pre-incubating the samples for 15 min with 1 µM of the specific ACE2-inhibitor DX600. The ACE2 activity is expressed as units per gram (U/g) protein for the cell lysate. One unit defines the amount of enzyme that hydrolyses 1 µmol of substrate per minute. Protein concentrations were determined via the Bradford method with BSA as the standard protein. Statistical analysis was performed using SPSS software version 27 (IBM) and graphs were designed with Graphpad Prism 9 software. Differences in activity in the cell lysate and supernatant between control and stimulated cells were assessed by the Kruskal-Wallis test.

No difference in ACE2 activity could be found between the control and stimulated cells (Figure S2.1). The ACE2 activity in the supernatant was below the limit of quantification.

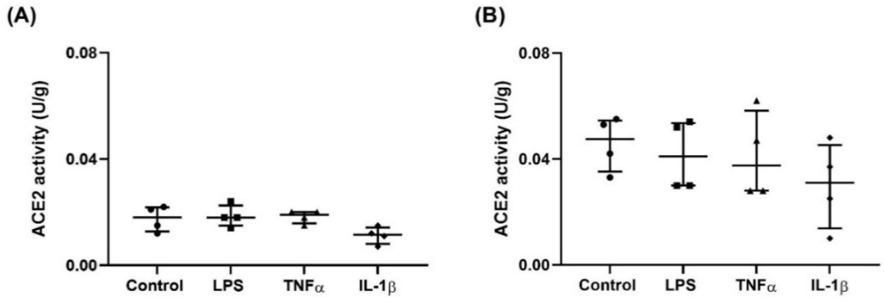


**Figure S2.1: ACE2 activity (U/g) in the cell lysate (A: HUVEC and B: HAoEC) after treatment with vehicle control, LPS, TNFα or IL-1β.** Results are reported as Median ± IQR. No difference was observed between the control and the stimulated groups (n=4-6; Kruskal-Wallis; p=0.098 for HUVEC; p=0.422 for HAoEC).
