# Supplementary material for: Prolyl Carboxypeptidase Mediates the C-Terminal Cleavage of (Pyr)-Apelin-13 in Human Umbilical Vein and Aortic Endothelial Cells
Source: Int J Mol Sci. 2021 Jun 22;22(13):6698. doi: 10.3390/ijms22136698 (PMC8268575; doi:10.3390/ijms22136698)
Supplement: Supplementary file 1 [file ijms-22-06698-s001.zip › Supplementary Material File 3.pdf]

Supplementary Material File S3. Cleavage by recombinant PREP

Recombinant human PREP, expressed in BL21 (DE3) cells as previously described [1], was pre-incubated for 15 min with 1 µM KYP-2047 (Laboratory for Medicinal Chemistry, University of Antwerp) or vehicle control at pH 7.4 (0.1 M potassium phosphate, 1 mM EDTA and 5 mM DTT). 100 µM substrate ((pyr)-apelin-13, (pyr)-apelin-$13_{(1\text{-}12)}$, Ang II or α-MSH 1-13) or vehicle control (PBS) was added. The reaction was stopped by acidification (pH<3) with 0.1% TFA after different time periods (0 s, 30 s, 1 min, 5 min, 15 min, 30 min or 1 h) and samples were stored at -80 °C.

To detect the cleavage of (pyr)-apelin-13 (m/z 1533.8), (pyr)-apelin-$13_{(1\text{-}12)}$ (m/z 1386.7), Ang II (m/z 1046.5) or α-MSH 1-13 (m/z 1665.8), the samples were analysed by MALDI-TOF/TOF. A search for product peaks of the different peptides was conducted.

No product peaks of (pyr)-apelin-13 or (pyr)-apelin-$13_{(1\text{-}12)}$ could be detected. Only the peaks corresponding with the intact peptides could be observed, indicating that recombinant PREP did not cleave (pyr)-apelin-13 or (pyr)-apelin-$13_{(1\text{-}12)}$. Ang II and α-MSH 1-13 were cleaved by rPREP at the C-terminus in function of time, as earlier reported. The cleavage of these substrates was abolished by 1 µM of KYP-2047, confirming the efficiency of the protocol.

1. Van Elzen, R.; Schoenmakers, E.; Brandt, I.; Van Der Veken, P.; Lambeir, A.M. Ligand-induced conformational changes in prolyl oligopeptidase: A kinetic approach. *Protein Eng. Des. Sel.* **2017**, *30*, 219–226, doi:10.1093/protein/gzw079.
