# Supplementary material for: Prolyl Carboxypeptidase Mediates the C-Terminal Cleavage of (Pyr)-Apelin-13 in Human Umbilical Vein and Aortic Endothelial Cells
Source: Int J Mol Sci. 2021 Jun 22;22(13):6698. doi: 10.3390/ijms22136698 (PMC8268575; doi:10.3390/ijms22136698)
Supplement: Supplementary file 1 [file ijms-22-06698-s001.zip › Supplementary Material File 4.pdf]

Supplementary Material File S4. Incubation of (pyr)-apelin-13 with cellular supernatant

HUVEC were seeded at a density of 10 000 cells per well in 96-well plates in 100 µL full medium. After 24 h, medium was replaced by assay medium and the cells were incubated for another 24 h at 37 °C in 5% CO_2. After 24 h, cell supernatants were harvested and incubated with vehicle control (1% DMSO) or 1 µM compound 8o for 15 min at 37 °C. Then, 100 µM (pyr)-apelin-13 or vehicle control (PBS) was added. The reaction was stopped by acidification (pH<3) with 0.1% TFA after 1 h and the samples were stored at -80 °C until further processing. The experiment was independently conducted 4 times. To detect the cleavage of (pyr)-apelin-13 (m/z 1533.8), the samples were analysed by MALDI-TOF/TOF.

(Pyr)-apelin-13 was cleaved at its C-terminus after 1 h and this cleavage was partly inhibited by compound 8o, indicating that PRCP was partially contributing to this cleavage (Figure S4.1). However, comparing this experiment with the initial experiment where (pyr)-apelin-13 was added to the cells for 24 h (Figure 1 of main manuscript), much less (pyr)-apelin-13 was converted here, indicating that secreted PRCP was not responsible for the major part of C-terminal (pyr)-apelin-13 cleavage seen in the initial experiment.

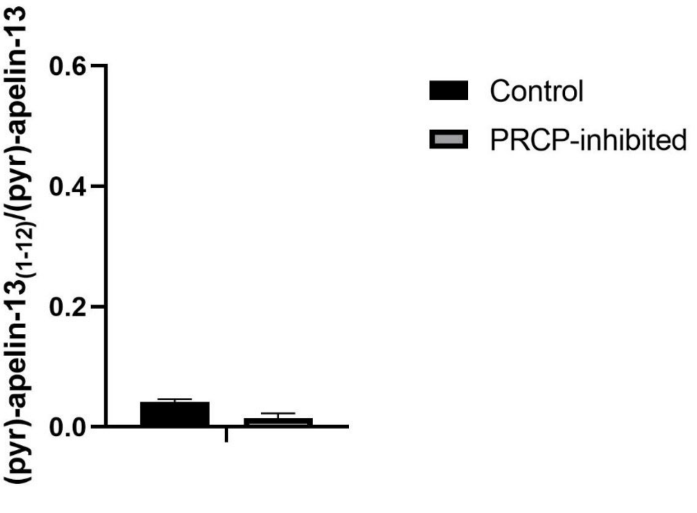

Figure S4.1: Incubation of 24 h cellular supernatant with (pyr)-apelin-13 during 1 h. (Pyr)-apelin-13 was cleaved at its C-terminus after 1 h and this cleavage was partly inhibited by compound 8o.
