# Supplementary material for: Prolyl Carboxypeptidase Mediates the C-Terminal Cleavage of (Pyr)-Apelin-13 in Human Umbilical Vein and Aortic Endothelial Cells
Source: Int J Mol Sci. 2021 Jun 22;22(13):6698. doi: 10.3390/ijms22136698 (PMC8268575; doi:10.3390/ijms22136698)
Supplement: Supplementary file 1 [file ijms-22-06698-s001.zip › Supplementary Material File 5.pdf]

Supplementary Material File S5. Viability assay

Viability of endothelial cells in presence of compound 8o or DX600 was determined using PrestoBlue® Cell Viability Reagent (Life Technologies) according to the manufacturer's instructions. HUVEC or HAoEC were seeded at 10 000 cells/well in 96-well plates in full medium. After 24 h, the cells were treated with vehicle control, 1 µM compound 8o or 1 µM DX600 (all final concentration of 1% DMSO) in assay medium for different time periods at 37 °C in 5% CO_2. PrestoBlue® reagent was added to the cells at a 1:10 ratio, followed by an incubation for 30 min at 37 °C. Hereafter, fluorescence at 570 nm was measured in an InfiniteTM 200 reader (Tecan). Viability of cells in the assay medium was first compared to viability of the cells in full medium.

The viability of endothelial cells cultured in assay medium compared with full medium is acceptable and stable over a period of 24 h (data not shown). As shown in Figure S5.1, 1 µM compound 8o or 1 µM DX600 had no effect on the cell viability of HUVEC or HAoEC, excluding any effects of inhibitor treatment due to cell death.

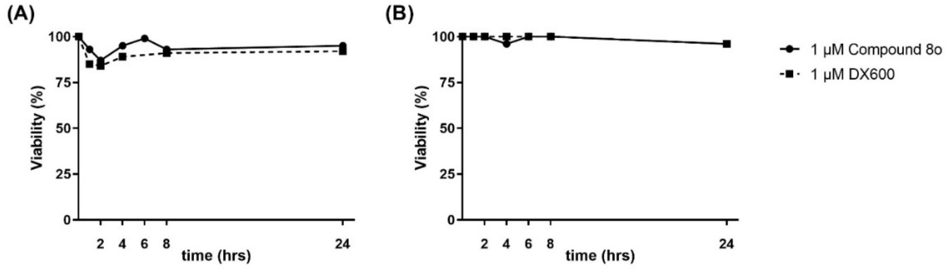

Figure S5.1: Viability (%) of HUVEC (A) and HAoEC (B) treated with 1 µM compound 8o or 1 µM DX600 in comparison with vehicle treated HUVEC measured with PrestoBlue® reagent.
