# Supplementary material for: Prolyl Carboxypeptidase Mediates the C-Terminal Cleavage of (Pyr)-Apelin-13 in Human Umbilical Vein and Aortic Endothelial Cells
Source: Int J Mol Sci. 2021 Jun 22;22(13):6698. doi: 10.3390/ijms22136698 (PMC8268575; doi:10.3390/ijms22136698)
Supplement: Supplementary file 1 [file ijms-22-06698-s001.zip › Supplementary Material File 6.pdf]

Supplementary Material File S6. Analysis of inhibitor potency

HUVEC and HAoEC were seeded at a density of $2.5x10^5$ cells/well in a 6 well plate. The next day, cells were treated with 1 µM compound 8o (final concentration of 1% DMSO) or 1 µM DX600 for 15 min or 24 h. After each time point, culture medium was harvested and stored at -80 °C for later activity measurements. Cells were harvested, washed with PBS and lysed for 1 h on ice in enzymatic activity lysis buffer (1% octyl glucoside, 70 µg/mL aprotinin, 10 mM EDTA, 50 mM Tris, pH 8.3) for PRCP and in the same lysis buffer without EDTA for ACE2.

As compound 8o and DX600 are reversible inhibitors, we followed the same procedure as previously described [1], to determine the residual enzymatic activity in the culture medium and the endothelial cell lysate. In brief, to estimate the residual enzymatic activity in biological samples of inhibitor-treated subjects/cells, a calibration curve was created with known concentrations of the inhibitor diluted in the biological sample that needs to be analysed. The percentage residual enzymatic activity was calculated by comparing enzymatic activity of inhibitor-treated cells with non-treated cells (defined as 100% activity).

In this case, we wanted to detect the residual enzymatic activity in the culture medium and the cell lysate of endothelial cells treated with compound 8o or DX600, so we created two calibration curves per inhibitor with known concentrations of inhibitor diluted in medium on the one hand and inhibitor diluted in lysis buffer on the other hand. Different concentrations (final concentrations: 0 nM, 0.49 nM – 500 nM) of compound 8o diluted in lysis buffer or medium were preincubated with rhPRCP for 15 min at 37 °C. Subsequently, Z-Pro-Phe (final concentration: 5 mM) was added and after 30 min incubation at 37 °C, stop solution (10% perchloric acid and 20% acetonitrile solution in purified water (v/v)) was added to stop the enzymatic reaction. The enzymatically formed Z-Pro was determined by its UV absorbance at 210 nm on a reversed phase HPLC (Shimadzu) and quantification was performed by peak height measurements[2]. Different concentrations (final concentrations: 0 nM, 0.49 nM – 500 nM) of DX600 diluted in lysis buffer or medium were preincubated with rhACE2 for 15 min at 37 °C. Subsequently, Mca-Ala-Pro-Lys(Dnp)-OH (Bachem, final concentration: 75 µM) was added and the release of Mca-Ala-Pro was measured kinetically with λ_{ex} = 320 nm and λ_{em} = 455 nm wavelengths for 20 min at 37 °C in an Infinite™ 200 reader (Tecan). The resulting enzymatic activity in these samples was stated as the percentage activity versus the non-inhibited sample. Since the inhibitors are reversible, the total dilution factor needs to be taken into account. However, when preparing the cell lysates an unknown dilution factor is introduced and the concentration calculated from the calibration curve will be inherently an estimation of

the actual intracellular inhibitor concentration. The calibration curves were fitted using a non-linear least squares method using GraFit 7 software, according to the following equation:

$$y = \frac{range}{1 + (\frac{x}{IC_{50}})^s} + background$$

where y is the ratio of the initial rate in presence and absence of inhibitor (v_i/v_0) expressed as a percentage, x is the concentration of the inhibitor in the assay, s is the slope factor and the IC_{50} is the half maximal inhibitory concentration. The fitted parameters of the calibration curve were used to estimate the percentage inhibition in the inhibitor-treated endothelial cells. To this effect, the enzymatic activity in the supernatant and the cell lysates must be measured in the same way as for the calibration curves. The residual activity in the samples was calculated as the difference between sample with exogenously added enzyme minus the sample without enzyme, this way correcting for background activity.

The calculations showed that in all samples PRCP was inhibited, meaning that incubation with 1 µM compound 8o for 15 min is sufficient to inhibit PRCP and to maintain PRCP-inhibition for 24 h in endothelial cells. ACE2 was inhibited in culture medium, meaning that incubation with 1 µM DX600 for 15 min is sufficient to fully inhibit ACE2 and to maintain full inhibition for 24 h in culture medium of endothelial cells. In the cell lysate, there was still residual activity. As ACE2 is a transmembrane protein with its catalytic site located extracellularly, there is no need for DX600 to enter the cell in order to inhibit ACE2.

1. Matheeussen, V.; Lambeir, A.M.; Jungraithmayr, W.; Gomez, N.; Mc Entee, K.; Van der Veken, P.; Scharpé, S.; De Meester, I. Method comparison of dipeptidyl peptidase IV activity assays and their application in biological samples containing reversible inhibitors. *Clin. Chim. Acta* **2012**, *413*, 456–462, doi:10.1016/j.cca.2011.10.031.

2. Kehoe, K.; Verkerk, R.; Sim, Y.; Waumans, Y.; Van Der Veken, P.; Lambeir, A.M.; De Meester, I. Validation of a specific prolylcarboxypeptidase activity assay and its suitability for plasma and serum measurements. *Anal. Biochem.* **2013**, *443*, 232–239, doi:10.1016/j.ab.2013.09.002.
