# Supplementary material for: Prolyl Carboxypeptidase Mediates the C-Terminal Cleavage of (Pyr)-Apelin-13 in Human Umbilical Vein and Aortic Endothelial Cells
Source: Int J Mol Sci. 2021 Jun 22;22(13):6698. doi: 10.3390/ijms22136698 (PMC8268575; doi:10.3390/ijms22136698)
Supplement: Supplementary file 1 [file ijms-22-06698-s001.zip › Supplementary Material File 7.pdf]

Supplementary Material File S7. Appropriate stimulation of HUVEC and HAoEC was confirmed by ELISA

IL-6 and IL-8 levels were measured in the supernatant of control and stimulated HUVEC and HAoEC by use of ELISA, according to the manufacturer's instructions (Immunotools). Appropriate stimulation of HUVEC and HAoEC was confirmed, since IL-6 levels were upregulated in the supernatant of IL-1β-stimulated cells and IL-8 levels in the supernatant of all stimulated cells in comparison with the control, according to previous observations [1,2].

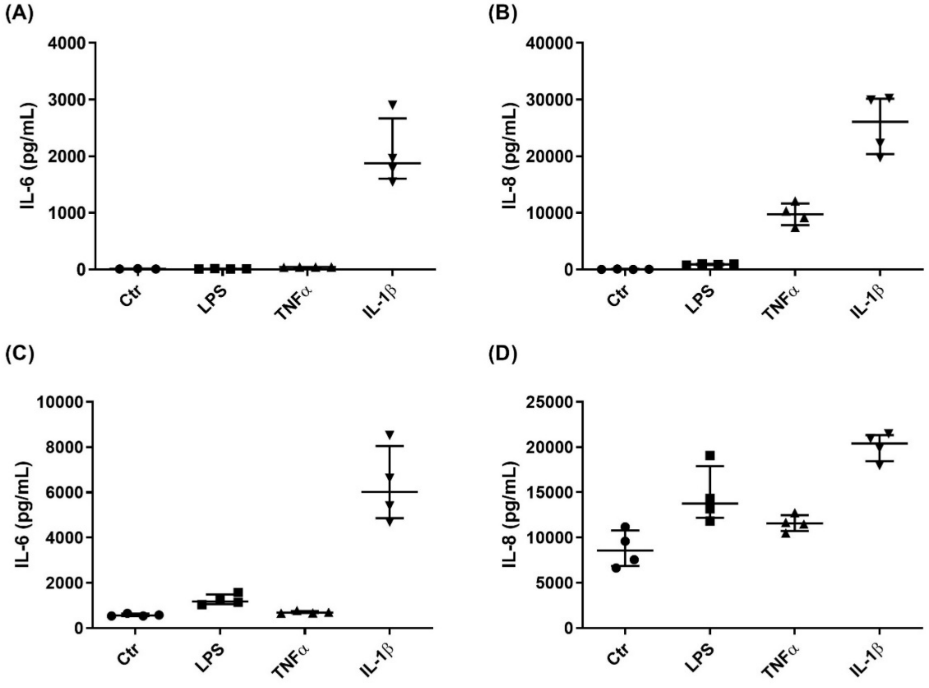

Figure S7.1: Appropriate stimulation of HUVEC and HAoEC was confirmed by ELISA. IL-6 and IL-8 levels in the supernatant of HUVEC (A,B) and HAoEC (C,D) after treatment with control, LPS, TNFα or IL-1β measured with ELISA (n=4). Results are reported as median ± IQR.

1. Makó, V.; Czúcz, J.; Weiszhár, Z.; Herczenik, E.; Matkó, J.; Prohászka, Z.; Cervenak, L. Proinflammatory activation pattern of human umbilical vein endothelial cells induced by IL-1β, TNF-α, and LPS. *Cytom. Part A* **2010**, *77*, 962–970, doi:10.1002/cyto.a.20952.

2. Kulhankova, K.; Kinney, K.J.; Stach, J.M.; Gourronc, F.A.; Grumbach, I.M.; Klingelhutz, A.J.; Salgado-Pabon, W. The Superantigen Toxic Shock Syndrome Toxin 1 Alters Human Aortic Endothelial Cell Function. *Infect. Immun.* **2018**, *86*, 1–16.
